# Development and evaluation of novel bio-safe filter paper-based kits for sputum microscopy and transport to directly detect *Mycobacterium tuberculosis* and associated drug resistance

**DOI:** 10.1371/journal.pone.0220967

**Published:** 2019-08-13

**Authors:** Divya Anthwal, Surabhi Lavania, Rakesh Kumar Gupta, Ajoy Verma, Vithal Prasad Myneedu, Prem Prakash Sharma, Hitesh Verma, Viveksheel Malhotra, Ashawant Gupta, Nalini Kant Gupta, Rohit Sarin, Sagarika Haldar, Jaya Sivaswami Tyagi

**Affiliations:** 1 Center for Bio-design and Diagnostics, Translational Health Science and Technology Institute, NCR Biotech Science Cluster, 3rd Milestone, Faridabad–Gurgaon Expressway, Faridabad, India; 2 Department of Biotechnology, All India Institute of Medical Sciences, Ansari Nagar, New Delhi, India; 3 Department of Microbiology, National Institute of Tuberculosis and Respiratory Diseases, Mehrauli, New Delhi, India; 4 TB Hospital, Ambala, India; 5 Advanced Microdevices Pvt Ltd, Industrial Area, Ambala Cantt, India; Jamia Hamdard, INDIA

## Abstract

India has the highest burden of Tuberculosis (TB) and multidrug-resistant TB (MDR-TB) worldwide. Innovative technology is the need of the hour to identify these cases that remain either undiagnosed or inadequately diagnosed due to the unavailability of appropriate tools at primary healthcare settings. We developed and evaluated 3 kits, namely ‘TB Detect’ (containing BioFM-Filter device), ‘TB Concentration and Transport’ (containing *Trans*-Filter device) and ‘TB DNA Extraction’ kits. These kits enable bio-safe equipment-free concentration of sputum on filters and improved fluorescence microscopy at primary healthcare centres, ambient temperature transport of dried inactivated sputum filters to central laboratories and molecular detection of drug resistance by PCR and DNA sequencing (Mol-DST). In a 2-site evaluation (n = 1190 sputum specimens) on presumptive TB patients, BioFM-Filter smear exhibited a significant increase in positivity of 7% and 4% over ZN smear and LED-FM smear (p<0.05), respectively and an increment in smear grade status (1+ or 2+ to 3+) of 16% over ZN smear and 20% over LED-FM smear. The sensitivity of Mol-DST in presumptive MDR-TB and XDR-TB cases (n = 148) was 90% for Rifampicin (95% confidence interval [CI], 78–96%), 84% for Isoniazid (95% CI, 72–92%), 83% for Fluoroquinolones (95% CI, 66–93%) and 75% for Aminoglycosides (95% CI, 35–97%), using phenotypic DST as the reference standard. Test specificity was 88–93% and concordance was ~89–92% (κ value 0.8–0.9). The patient-friendly kits described here address several of the existing challenges and are designed to provide ‘Universal Access’ to rapid TB diagnosis, including drug-resistant disease. Their utility was demonstrated by application to sputum at 2 sites in India. Our findings pave the way for larger studies in different point-of-care settings, including high-density urban areas and remote geographical locations.

## Introduction

Tuberculosis (TB) remains a deadly killer with more deaths than HIV and malaria combined [1]. According to World Health Organization (WHO) estimates, there were 10 million new TB cases with 1.3 million TB deaths worldwide in 2017, of which 3.6 million cases were missed due to a lack of diagnosis [1]. The large number of cases that are missed in diagnosis and the alarming increase in drug resistant TB infection has created a critical need for near-patient and cost-effective technologies that can rapidly detect *Mycobacterium tuberculosis* (*M*. *tuberculosis*) and associated drug resistance.

Despite the low and variable sensitivity of direct smear microscopy that ranges between 20 to 60% [2], this rapid and inexpensive technique is the most frequently used diagnostic test for pulmonary TB in peripheral or primary healthcare centres (PHCs and associated Designated Microscopy Centres [DMCs]), especially in resource-constrained settings [3]. The sensitivity of direct smear microscopy was enhanced 6% by the use of LED-based fluorescent microscopy [4]; moreover, several efforts have been made to increase the sensitivity of smear microscopy by sputum concentration through centrifugation [5]. Recently, a vacuum pump-based small membrane filtration (SMF) method was reported for visualizing *M*. *tuberculosis* on a membrane [6]. However, the SMF method required bio-safety laboratory facilities and its diagnostic yield remained comparable to that of conventional fluorescence microscopy [7].

Numerous Nucleic Acid Amplification Tests (NAATs) are endorsed by WHO, namely Xpert MTB/RIF (Xpert) [8] and Xpert Ultra [9] (for rifampicin [RIF] resistance detection), Line Probe Assays (LPAs) (for both MDR-TB and extensively drug resistant [XDR]-TB detection) [10, 11] and Loopamp MTBC (LAMP) assay [12]. However, these rapid molecular assays are restricted to National/Intermediate reference laboratories (NRLs/IRLs) and only the LAMP assay is intended for use at microscopy centres [13]. Recently, a chip-based real-time PCR assay i.e. Truenat was developed for the detection of TB and RIF resistant TB [14]. However it does not have enough evidence for a WHO endorsement at present [13]. Sequencing has also emerged as a powerful tool for detecting drug resistance at central laboratories [15], however evidence for its large scale utility for drug resistance detection directly from clinical specimens such as sputum is still being generated [1].

The widespread implementation of molecular tests for drug resistance determination is a challenge as it requires transportation of potentially infectious sputum at low temperatures using bio-safe containers from PHCs and associated DMCs to central laboratories (NRL/ IRL). Some commercially available products for sputum transport have been developed, such as OMNIgene SPUTUM [16–18], PrimeStore MTM [19] and FTA card or Geno card [20], which await further testing [21]. In addition, in resource-constrained settings where access to BSL-3 level laboratories is limited, the safety of laboratory personnel who handle infectious clinical material is a major concern [3].

To address these diagnostic challenges, 3 kits namely, ‘TB Detect’, ‘TB Concentration & Transport’ and ‘TB DNA Extraction’ kits were developed that improve the sensitivity of smear microscopy and enable biosafe transport of sputum at ambient temperature for extraction of *M*. *tuberculosis* DNA suitable for downstream applications of targeted sequencing for the detection of TB, MDR-TB and XDR-TB.

## Materials & methods

### Ethical clearance

The entire study was performed using fresh sputum samples which were collected between August 2016 to August 2017 after obtaining ethical clearance from the Institutional Ethics Committees of National Institute of Tuberculosis and Respiratory Diseases (NITRD, EC/LRS/2013/2543), TB Hospital, Ambala (BIRAC/2015/9), All India Institute of Medical Sciences (AIIMS, IEC/NP-135/2013) and Translational Health Science and Technology Institute (THSTI, THS 1.8.1 [14]). We obtained written informed consent from participants or parents (in case of minors) in accordance with ethical guidelines from participating institutions. (S1 Appendix). Our study adheres to the Standards for Reporting of Diagnostic Accuracy (STARD) guidelines and a completed checklist is included (S2 Appendix).

### Salient features of developed kits

**Fig 1:**
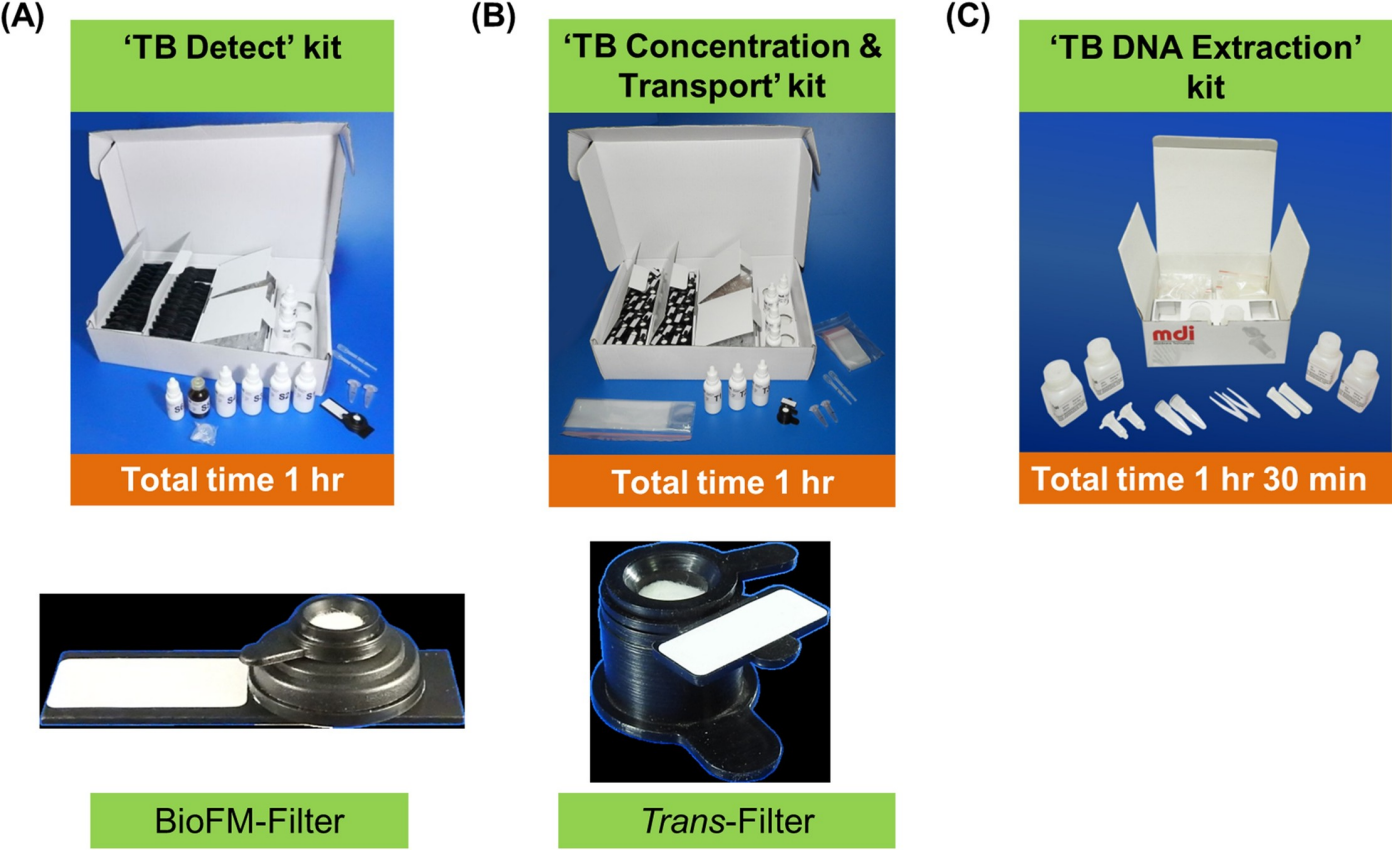
Developed kits: (A) ‘TB detect’ kit; (B) ‘TB concentration & transport’ kit; and (C) ‘TB DNA extraction’ kit.

In brief, the kit technology consists of liquefying the sputum and concentrating the bacteria in sputum on a membrane by use of a filtration device. This filter technology is unique in that the bacteria are retained on the filter during passage of liquefied sputum through it by gravity and absorbent pad-based capillary action, which pull the liquid across the filter and eliminate the need for any external vacuum or pressure-creating equipment. A removable pre-filter funnel is included which retains larger particles and mucus-like components of the sputum, but allows bacteria to pass through the pre-filter. ‘TB Detect’ kit uses a black non-fluorescent membrane and black non-fluorescent plastic device (BioFM-Filter device) to provide good contrast and easy detection of bacteria. *In-situ* fluorescent staining of the bacteria reduces the process time dramatically. In ‘TB Concentration & Transport’ kit, the bacteria-laden membrane is easily removable from the *Trans*-Filter device and used to safely transport the bacteria in a dry manner by envelope to central laboratories for molecular diagnostics (Fig 1). On-device sample processing using *Trans*-Filter enables bio-safe ambient temperature transport of filter, stabilizes the bacteria on the filter and also rids the sample of potential inhibitors of DNA tests.

Briefly, the ‘TB Detect’ and ‘TB Concentration & Transport’ kits contain ‘Dissolving solution’ to decontaminate and liquefy the sputum for filtration through the BioFM-Filter and *Trans*-Filter. The final composition of ‘Dissolving solution’ (Guanidinium Hydrochloride, Tris buffer, Triton X-100 and reducing agent of functionality similar to DTT) was derived after optimizing (i) sputum liquefaction and compatibility of the solution with BioFM-Filter and *Trans*-Filter, (ii) bacterial visualization on BioFM-Filter, (iii) bacterial retention on *Trans*-Filter and (iv) reagent stability at room temperature. Various types of sputum (thick, mucopurulent, purulent, mucoid and saliva) were used during kit optimization. BioFM-Filter and *Trans-*Filter devices were co-developed with ‘Dissolving solution’ and reiteratively optimized for various parameters (S3 Appendix). Briefly, the BioFM-Filter was assessed for *in-situ* staining and microscopy, while the *Trans-*Filter device was optimized for bacterial retention, stability during transportation and compatibility with the isolation of PCR-amplifiable DNA (S3 Appendix). Finally, all the components including the ‘Filter device’, ‘Dissolving solution’, other solutions and supplies were assembled into a kit format (Fig 1). The third kit, ‘TB DNA Extraction’ kit, enabled DNA extraction from the transported filter based on the principle of silica column-based binding and elution using Tris-EDTA (TE) buffer. Various combinations of solutions and columns were checked for their ability to provide ultra-pure *M*. *tuberculosis* DNA compatible with PCR amplification and targeted sequencing (referred as Molecular Drug Susceptibility [Mol-DST] assay hereafter).

### Bio-safety evaluation

**Fig 2:**
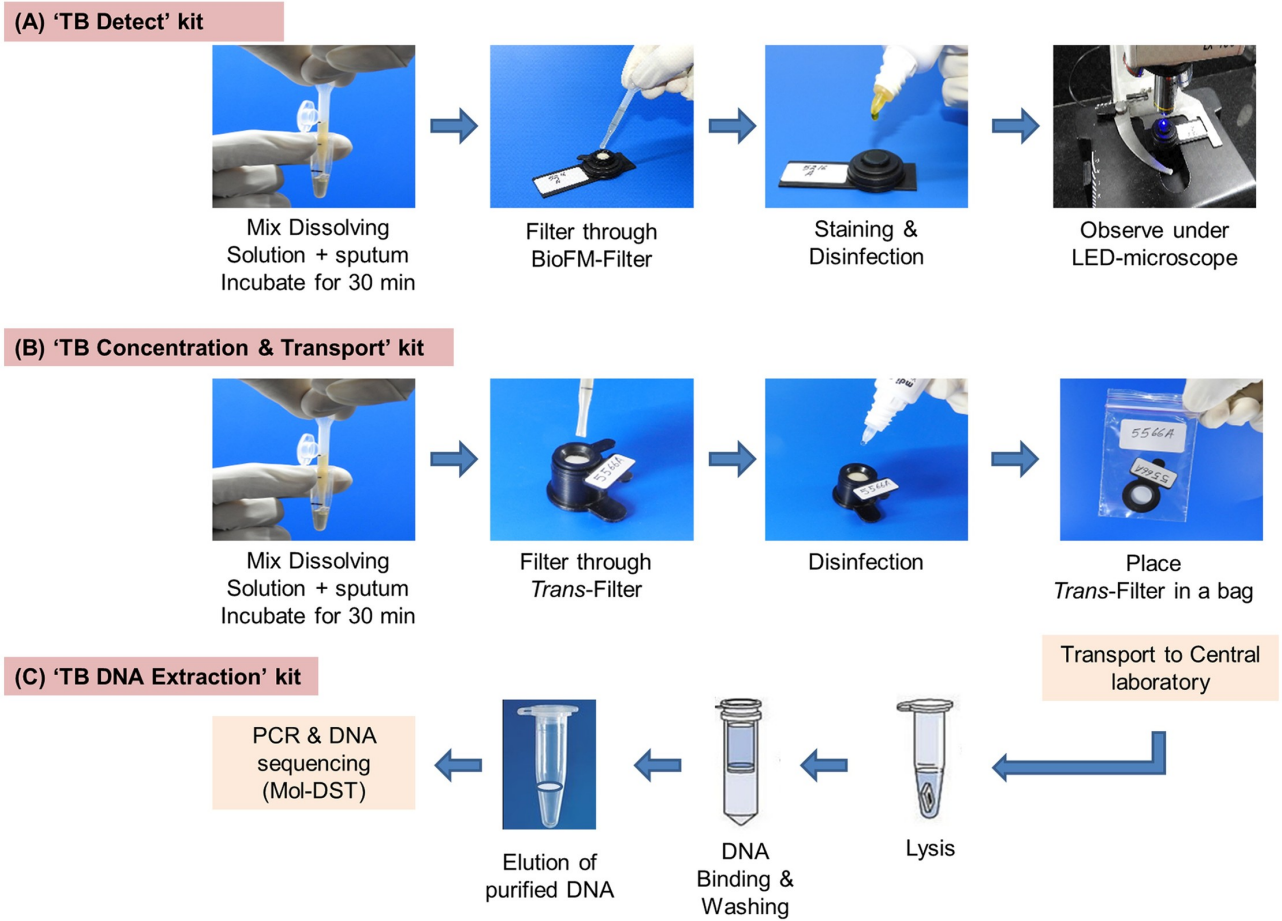
(A) Sample processing and staining of BioFM-Filter by using ‘TB detect’ kit; (B) sample processing by using ‘TB concentration & transport’ kit; (C) DNA extraction from *Trans-*Filter by using ‘TB DNA extraction’ kit.

The ‘TB Detect’ and ‘TB Concentration & Transport’ kits were assessed for sputum disinfection in a dedicated BSL 2+ laboratory at AIIMS. In this experiment performed in duplicates, *M*. *tuberculosis* bacteria (ranging from 10^3^ to 10^8^ bacteria) were spiked into 1 ml of smear-negative sputum and processed using ‘TB Detect’ and ‘TB Concentration & Transport’ kits (Fig 2A and 2B). Control spiked sputum was processed in parallel without bio-safety treatment. The ‘TB DNA Extraction’ kit was used to isolate mycobacterial DNA from bio-safe *Trans*-Filter after sputum processing (Fig 2C) and has no role in bio-safety processing; therefore, bio-safety experiment was not performed for it.

**Fig 3:**
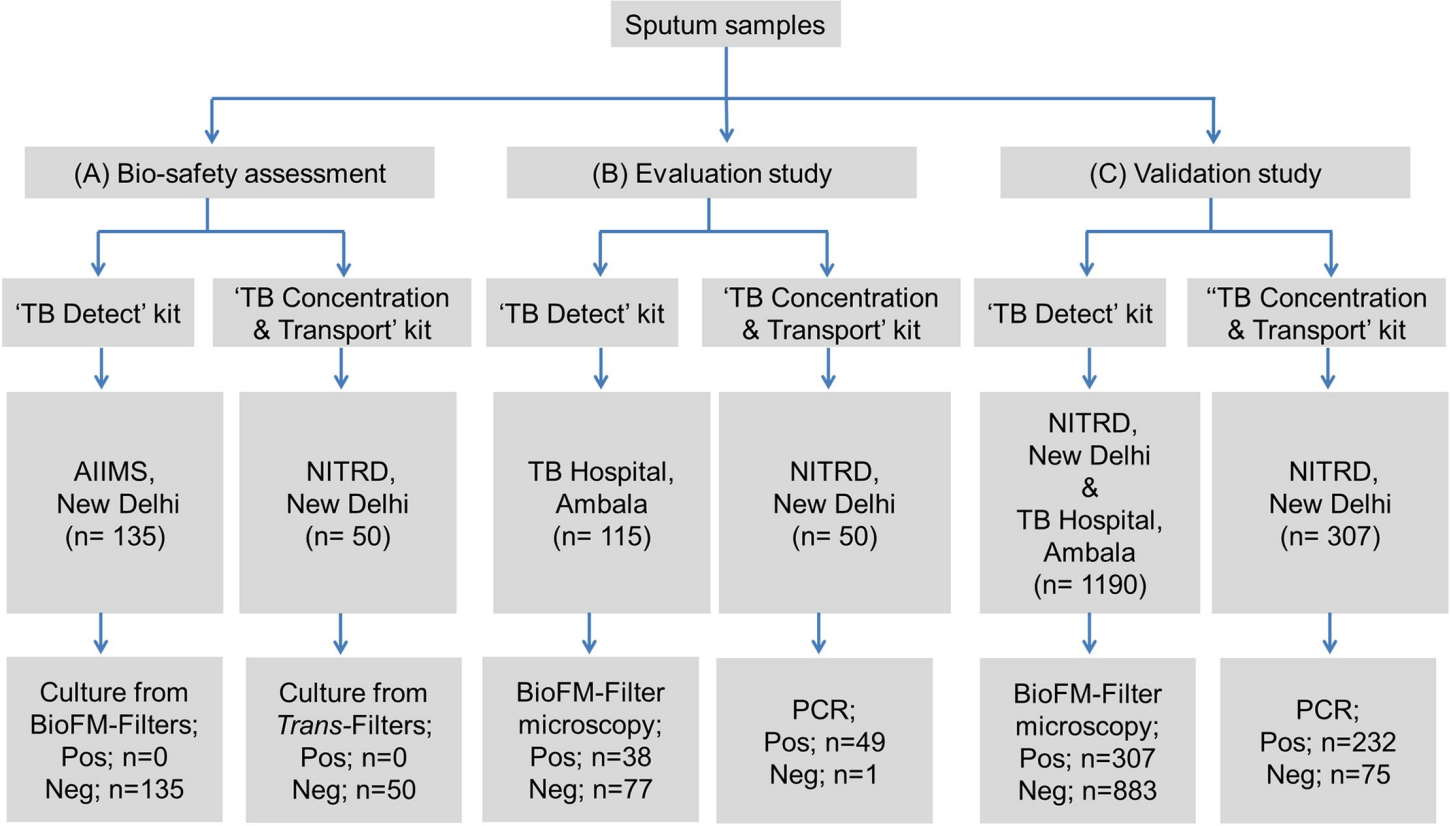
Study design.

Smear-positive sputum from presumptive TB patients (n = 135) and presumptive MDR-TB patients (n = 50) were assessed (Fig 3A). Processed BioFM-Filters and *Trans*-Filters were washed with neutral pH buffer and were placed in 7H9 liquid culture media [containing albumin dextrose complex with PANTA (polymyxin B, amphotericin B, nalidixic acid, trimethoprim and azlocillin) supplement (Becton Dickinson)]. The tubes were incubated at 37 ºC for up to 8 weeks and observed for growth, which was subsequently confirmed by Ziehl Neelsen (ZN) smear and SD BIOLINE TB Ag MPT64 Rapid test.

### Assessment of bacterial stability on filter

Sputum samples of all grades (3+ to scanty) were processed using ‘TB Concentration & Transport’ kit (5 filter devices/grade) to assess the effect of temperature during transportation of *Trans*-Filter. One *Trans*-Filter of each sample grade was processed on the same day using ‘TB DNA Extraction’ kit and the remaining 4 *Trans*-Filters (individually packed) were incubated at 50ºC and processed at weekly intervals for upto 4 weeks to mimic high ambient summer temperatures in India that ranges between 37 ºC to 50 ºC (S3 Appendix). The quality of DNA isolated from the filter was then assessed by the Mol-DST assay (see below).

### Limit of detection (LOD)

The LOD of ‘TB Detect’ and ‘TB Concentration & Transport’ kits were determined by spiking *M*. *tuberculosis* bacteria (H37Rv) into sputum sample. *M*. *tuberculosis* bacteria were spiked into smear-negative sputum in a range of 100 to 5000 bacteria in 1 ml of sputum. Six independent series of spiked sputum were processed with ‘TB Detect’ kit and then BioFM-Filter was observed under LED-FM microscope (40x magnification) (Fig 2A). For ‘TB Concentration & Transport’ kit, 2 independent series of spiked sputum (in duplicates) were processed and then DNA was extracted from *Trans*-Filter with ‘TB DNA Extraction’ kit according to the kit protocol and assessed by the Mol-DST assay (Fig 2B and 2C, S3 Appendix). These LOD experiments were performed in the BSL 2+ laboratory at AIIMS.

### Mol-DST assay

Mol-DST comprises of a PCR assay followed by targeted DNA sequencing. The primers used in the Mol-DST assay were based on the most frequently reported mutations for resistance to TB drugs. Mol-DST targets the genes *rpoB*, *katG*, *mabA*-*inhA* promoter region, *gyrA* and *rrs* responsible for 70–95% of all drug resistance [22–26].

### Evaluation study

The ‘TB Detect’ kit was evaluated on 115 prospectively collected fresh sputum samples from presumptive TB cases at TB Hospital, Ambala (Fig 3B). All the samples were processed with ‘TB Detect’ kit and the results of BioFM-Filter microscopy were compared with that of direct ZN and LED-FM microscopy performed simultaneously by standard procedures [27]. The ‘TB Concentration & Transport’ and ‘TB DNA Extraction’ kits were evaluated on 50 sputum samples of presumptive MDR-TB/XDR-TB cases at NITRD, New Delhi (Fig 3B). All samples were collected and processed by ‘TB Concentration & Transport’ kit at NITRD Hospital and the *Trans-*Filters were transported to THSTI for DNA extraction and Mol-DST assay.

### Validation of kits

#### Selection of subjects

**Fig 4:**
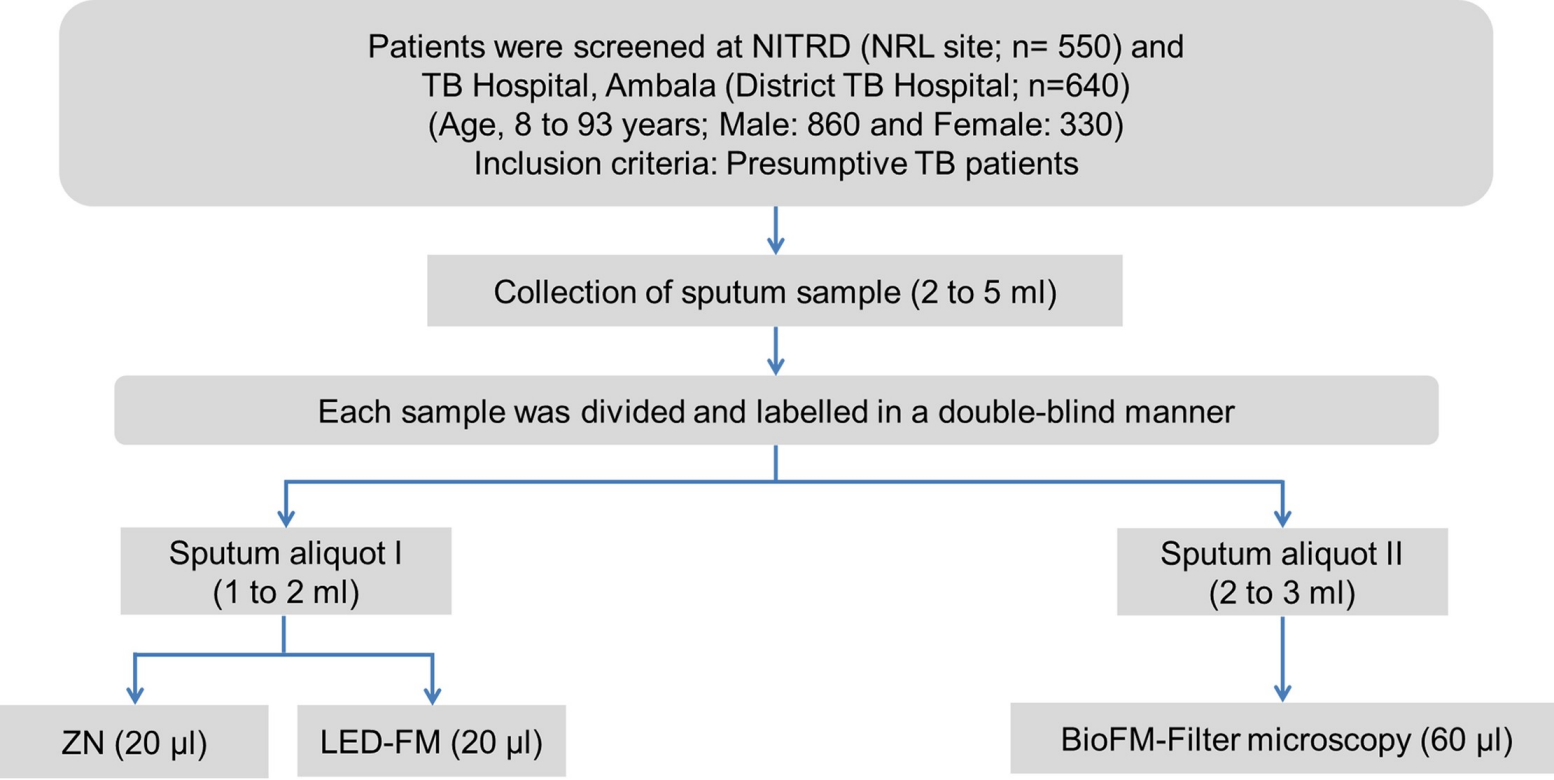
Workflow of the ‘TB detect’ kit evaluation study.

**Fig 5:**
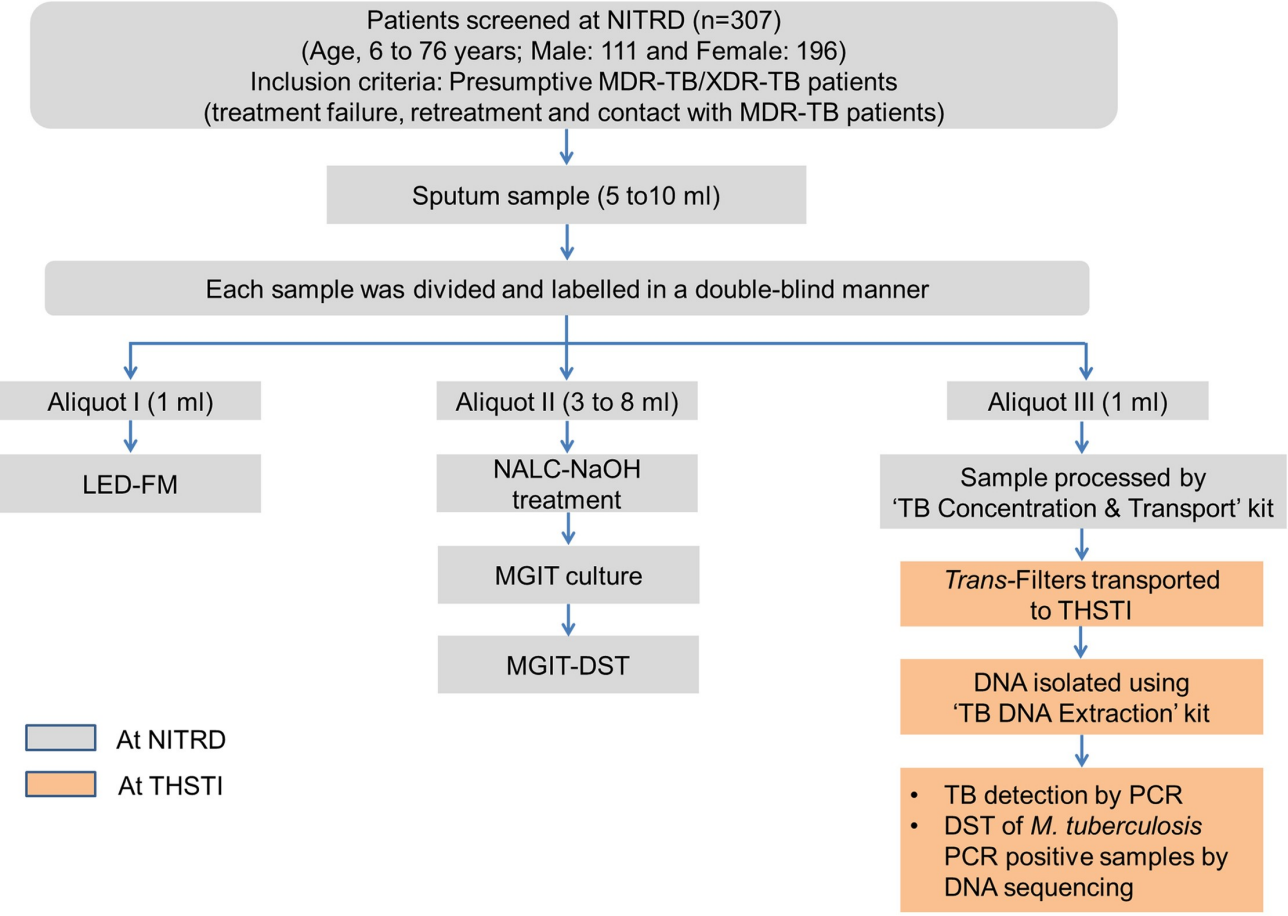
Workflow of the ‘TB concentration & transport’ kit and ‘TB DNA extraction’ kit evaluation study.

The ‘TB Detect’, ‘TB Concentration & Transport’ and ‘TB DNA Extraction’ kits were validated in a double-blind study. ‘TB Detect’ kit was validated on prospectively collected sputum samples of presumptive TB patients attending the Outpatient Department between August 2016 and March 2017 at two sites: NITRD, New Delhi (a National Reference Laboratory, n = 550) and TB Hospital, Ambala (a district TB Hospital, n = 640) (Figs 3C and 4, S1 Table). The validation of ‘TB Concentration & Transport’ and ‘TB DNA Extraction’ kits was undertaken on sputum samples of prospectively enrolled presumptive MDR-TB/XDR-TB patients (treatment failure and retreatment patients or in contacts of MDR-TB patients) enrolled between September 2016 and August 2017 at NITRD, New Delhi (n = 307, Figs 3C and 5, S1 Table).

### Validation study design

**‘TB Detect’ kit:** Sputum samples were processed as described below for ZN, LED-FM and ‘TB Detect’ kit-based BioFM-Filter microscopy (Fig 4).

**Direct ZN and LED-FM smear microscopy:** For direct smear microscopy, a loopful (5 mm, 20 μl) of sputum sample was taken in duplicate for smear preparation of 2 by 3 cm, air-dried and heat fixed. One slide was processed for ZN staining and the second slide was stained with auramine O and the slides were observed under the light microscope (100x magnification) and LED microscope (40x magnification, LaboMed LX-200 LED microscope, Ambala, India), respectively. The smear grading of sputum was done according to standard Revised National TB Control Program (RNTCP) guidelines [27].

**Sputum processing and smear microscopy by ‘TB Detect’ kit:** Aliquot II of all sputum samples was processed by the ‘TB Detect’ kit, according to the kit protocol (Fig 2A). Briefly, 400 μl of ‘Dissolving solution’ (S1) was added to 100 μl of sputum in a ratio of 4:1 (v/v) and incubated for 30 minutes. Thereafter, 300 μl out of 500 μl of liquefied sputum (~ to 60 μl of neat sputum) was filtered through the BioFM-Filter and *in-situ* fluorescence staining (using S2 to S6 solutions) was performed using the standard RNTCP protocol. BioFM-Filter was viewed at 40× magnification under a LED microscope (LaboMed LX-200 LED microscope) and the smears were graded according to standard RNTCP guidelines for LED-FM [27].

**‘TB Concentration & Transport’ and ‘TB DNA Extraction’ kits:** Sputum samples were processed as described (Fig 5). Aliquot I was used to perform LED-FM microscopy as described above and Aliquot II for Mycobacterial Growth Indicator Tube (MGIT) culture. Further phenotypic MGIT-DST was performed on positive cultures. Aliquot III was used for testing the ‘TB Concentration & Transport’ kit and ‘TB DNA Extraction’ kit (Fig 2B and 2C). Briefly, the *Trans*-Filters were transported to THSTI and DNA was extracted from *Trans*-Filters by using the ‘TB DNA Extraction’ kit. The extracted DNA was used to perform the Mol-DST assay (PCR and DNA sequencing).

**MGIT Culture:** Aliquot II was decontaminated by the NALC-NaOH method [28]. Briefly, 1:1 volume of NALC-NaOH solution was added to each sputum sample, vortexed and incubated for 15 min at room temperature followed by addition of phosphate buffer saline (PBS) and centrifuged at 3000 x g for 15 min at room temperature. The supernatant was discarded and the pellet was resuspended in 2 to 3 ml PBS [29]. Then, 0.5 ml of the resuspended pellet was added to the MGIT tube containing 0.8 ml PANTA supplement (BD Microbiology System) and incubated up to 42 days [30]. The growth of bacteria belonging to *M*. *tuberculosis* complex was confirmed by using ZN smear and SD BIOLINE TB Ag MPT64 Rapid test (Standard Diagnostics).

**MGIT-DST:** Phenotypic DST was performed from positive *M*. *tuberculosis* cultures for two first line drugs, RIF (1.0 μg/ml) and INH (0.1 μg/ml) and for second line drugs Kanamycin (KAN, 2.5 μg/ml), amikacin (AMK, 1.0 μg/ml), capreomycin (CAP, 2.5 μg/ml) and ofloxacin (OFL, 2.0 μg/ml) with the use of the BD BACTEC MGIT 960 automated mycobacterial detection system [30–32].

**Sputum processing and DNA extraction using ‘TB Concentration & Transport’ and ‘TB DNA Extraction’ kits:** Aliquot III of all sputum samples was processed by the ‘TB Concentration & Transport’ kit according to the kit protocol (Fig 2B). Briefly, ‘Dissolving solution’ (T1) was added to 100 μl of sputum in a ratio of 4:1 (v/v) and incubated for 30 minutes. Thereafter, 300 μl liquefied sputum (equivalent to ~60 μl of neat sputum) was added to the *Trans*-Filter followed by the addition of phenol-based ‘Sterilizing solution’ (T2, pH- 5.5) and neutral pH buffer i.e. ‘Stabilizing solution’ (T3). After transport, DNA was extracted from the *Trans*-Filter by using the ‘TB DNA Extraction’ kit involving four steps: lysis, binding, washing and elution (Fig 2C). The DNA was extracted from filter in 100 μl elution buffer and used for performing Mol-DST.

**Molecular Drug Susceptibility (Mol-DST) Testing:** The extracted DNA was used to amplify the resistance-determining regions of MDR-TB and XDR-TB markers (*rpoB*, *katG*, *inhA*, *gyrA* and *rrs*) by PCR as reported earlier [33, 34]. The inhibitory effect of filter material, if any, on PCR amplification was excluded by PCR inhibition check reactions. The PCR products were shipped to Thermo Fisher Scientific, Gurgaon, India for DNA sequencing and the results were analysed by BioEdit Sequence Alignment Editor software ver. 7.2.5 by using *M*. *tuberculosis* H37Rv sequence as a reference and ABI SeqScanner software-2 ver. 2.0 for electropherogram analysis.

**Kit performance and statistical analysis:** One objective of the study was to evaluate the performance of the BioFM-Filter microscopy using ‘TB Detect’ kit against direct ZN and LED-FM microscopy. Test positivity was calculated as [Total positives]/ [Total number of samples] and the Chi square test was used to compare the positivity increment of ‘TB Detect’ over ZN and LED-FM using GraphPad Prism version 6.00 for Windows, (GraphPad Software, La Jolla California USA, www.graphpad.com). The concordance between two tests (‘TB Detect’ vs. LED-FM/ ZN) was calculated as [positive by both tests + negative by both tests] / [total number of samples] and the degree of agreement was quantified by Cohen’s kappa (κ) (https://www.graphpad.com/quickcalcs/kappa1/). The second objective was to evaluate the utility of ‘TB Transport’ (containing *Trans*-Filter) and ‘TB DNA Extraction’ kits for sputum transport, *M*. *tuberculosis* DNA isolation and Mol-DST assay, taking phenotypic DST as the reference standard. The sensitivity of Mol-DST assay was calculated as [True positives] / [True positives + False negatives]; wherein true positives are defined as samples identified as resistant by both the Mol-DST assay and phenotypic DST, and false negatives are samples which were missed by the Mol-DST assay but showed resistance by phenotypic DST. Specificity was defined as [True negatives] / [True negatives + false positives]; where true negatives are defined as samples sensitive by both Mol-DST and phenotypic DST and false positives are samples showing mutations by Mol-DST but sensitive by phenotypic DST. Concordance between Mol-DST and phenotypic DST results was calculated as [True positives + true negatives] / [total number of samples] [35]. The degree of concordance/agreement was measured by Cohen’s kappa (κ) as described [36].

## Results

The kits developed in the study are all-inclusive; the ‘TB Detect’ kit contains BioFM-Filter and all the supplies required for sputum processing and *in-situ* staining on filter for microscopy; the ‘TB Concentration & Transport’ kit includes *Trans*-Filter with all supplies for sputum processing and transport while the ‘TB DNA Extraction’ kit includes all supplies for DNA extraction from *Trans*-Filter (Figs 1 and 2).

### Bio-safety check, LOD and bacterial stability on filter

**Fig 6:**
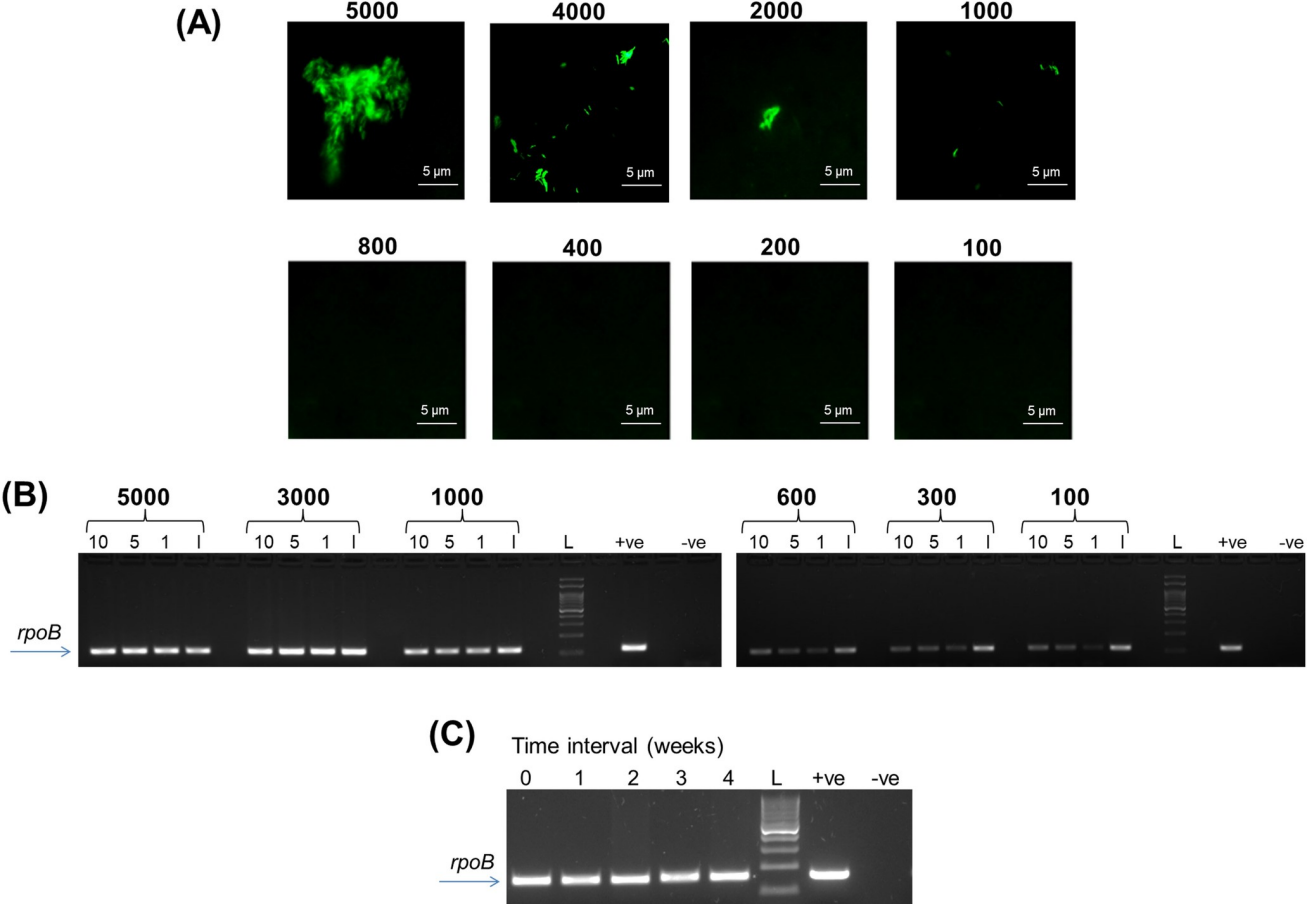
(A) Limit of Detection of BioFM-Filter microscopy (40x magnification); (B) PCR amplification of DNA isolated from *Trans*-Filter. 10, 5, 1 indicate the amount of DNA (in μl) added in PCR and I indicates inhibitor check reaction. Numbers in panels (A) and (B) indicate the number of *M*. *tuberculosis* bacteria spiked in 1 ml of sputum; (C) Assessment of stability of DNA on *Trans-*Filter. Well 0 represents amplification of freshly isolated DNA (day 0) and wells 1 to 4 represent amplification of DNA isolated from *Trans-*Filters stored at 50ºC at weekly intervals upto 4 weeks. Data for a scanty smear grade sputum sample is shown.

*M*. *tuberculosis*-spiked sputum samples (10^3^ to 10^8^ bacteria in 1 ml of smear-negative sputum) did not exhibit bacterial growth in liquid culture. The absence of growth was confirmed by ZN microscopy and SD BIOLINE TB Ag MPT64 Rapid test. Next, the effectiveness of the ‘TB Detect’ and ‘TB Concentration & Transport’ kits was confirmed in smear-positive sputum derived from presumptive TB subjects (n = 135, Fig 3A) and from presumptive MDR-TB patients (n = 50, Fig 3A), respectively. None of the kit-processed samples yielded growth in liquid media (S2 Table). The LOD of BioFM-Filter microscopy using ‘TB Detect’ kit was estimated to be 1000 bacteria/ml and that of ‘TB Concentration & Transport’ kit and ‘TB DNA Extraction’ kit was 100 bacteria/ml (Fig 6A and 6B, S3 Appendix).

*Trans*-Filter of ‘TB Concentration & Transport’ kit was compatible with the isolation of PCR-amplifiable DNA (Fig 6C and S3 Appendix) and subsequent Mol-DST. *Trans*-Filter was incubated for upto 4 weeks at 50°C to simulate high ambient temperatures that prevail during the summer months in India. PCR amplification efficiency and quality of the sequencing data (read length, minimum background noise and ‘a good base call’) from the isolated DNA were not compromised by these conditions and thereby demonstrated the robustness of the filter-based sputum transport device.

### Evaluation study

The performance of the ‘BioFM- Filter’ were compared with those of ZN and LED-FM microscopy (n = 115) that were performed simultaneously on the same sputum samples (Fig 3B). The overall positivity of ‘BioFM-Filter’ was 33% as compared to 18% and 22% in ZN and LED-FM smear microscopy, respectively. Importantly, the ‘BioFM-Filter’ picked up 14 additional samples as positive that were missed by ZN as well as LED-FM microscopy.

The ‘TB Concentration & Transport’ and ‘TB DNA Extraction’ kits were evaluated on 50 sputum samples of presumptive MDR-TB/XDR-TB cases to assess their suitability for Mol-DST (Fig 3B). DNA was extracted from all the transported *Trans*-Filters (DNA concentration ranged between 2.3 to 120 ng/μl in 100 μl elution buffer) and was inhibitor-free, amplifiable by PCR for resistance-determining regions of MDR-TB and XDR-TB markers and compatible with targeted DNA sequencing (Mol-DST assay).

### Validation of kits

#### ‘TB Detect’ kit

**Fig 7:**
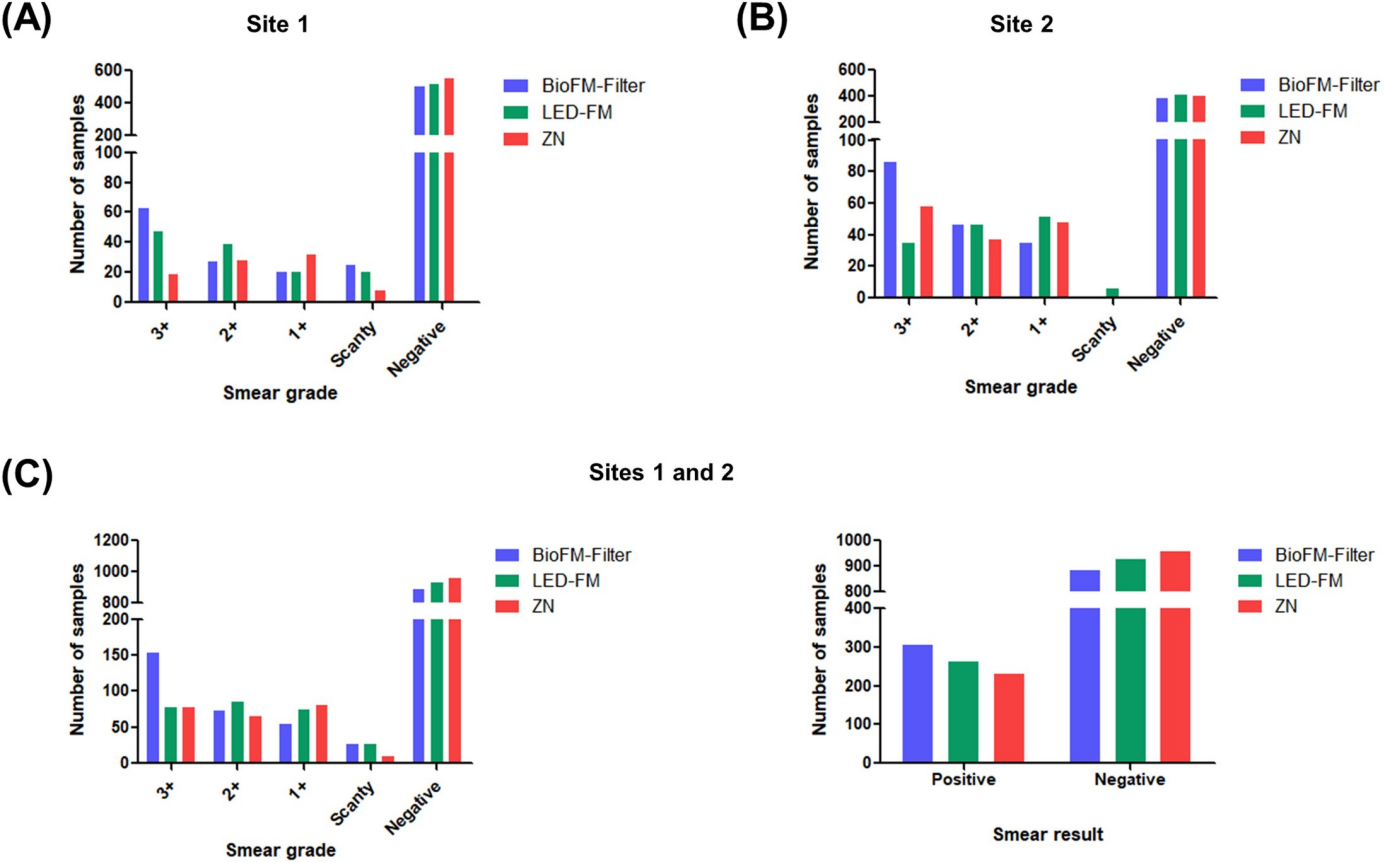
Comparison of smear grade status by ‘BioFM-Filter’ vs. Direct smear microscopy (LED-FM and ZN). (A) At site 1, NITRD (n = 550); (B) At site 2, TB hospital, Ambala (n = 640); (C) combined performance at both the sites (n = 1190). Left panel: smear grade status; right panel: smear results.

Based on the encouraging performance of the kit in the evaluation study, a double-blind validation of ‘TB Detect’ kit was performed at NITRD and TB Hospital, Ambala (n = 1190, Figs 3C and 4). ‘BioFM-Filter’ microscopy showed an overall positivity of 26% (307/1190) as compared to ZN smear (19%; 231/1190) and LED-FM smear (22%; 263/1190), with an increment of 7% and 4%, respectively (Table 1 and Fig 7). The increment in positivity of BioFM-Filter was significant over ZN and LED-FM (p<0.05), however, the positivity increment of LED-FM over ZN was not found to be significant (p value = 0.12). An increment of 16% and 20% was found in grade status from 1+ or 2+ observed in ZN direct smear and LED-FM direct smear, respectively, to 3+ by BioFM-Filter microscopy (Fig 7). The concordance between BioFM-Filter microscopy vs. LED-FM was 93% and the degree of agreement (κ) was 0.8 (Standard error [SE] of kappa- 0.02 and 95% CI- 0.77 to 0.85). The concordance between BioFM-Filter microscopy vs. ZN was 90.8% and the degree of agreement (κ) was 0.73 (SE of kappa- 0.02 and 95% CI- 0.69 to 0.78).

**Table 1:** Performance of ‘BioFM-filter’, LED-FM and ZN microscopy.

Microscopy	Site 1 NITRD Hospital (n = 550)		Site 2 TB Hospital, Ambala (n = 640)		Total (n = 1190)	
	Pos (% Positivity)	Neg	Pos (% Positivity)	Neg	Pos (% Positivity)*	Neg
BioFM-Filter	168 (30)	382	139 (21)	501	307 (26)	883
LED-FM	138 (25)	412	125 (19)	515	263 (22)	927
ZN	144 (26)	406	87 (13)	553	231 (19)	959

Pos, Positive; Neg, Negative

*Positivity increment of BioFM-Filter vs. ZN and LED-FM was significant (p<0.05) and LED-FM vs. ZN was not significant (p value = 0.12).

At Site 1, 127 of 550 samples were positive by all the three tests. BioFM-Filter microscopy yielded 131 positive results when combined with ZN microscopy and 133 positives when combined with LED-FM microscopy, as compared to 129 positives obtained when ZN and LED-FM microscopy results were combined (S3 Table). Individually, BioFM-Filter microscopy detected 37 samples that were missed by ZN microscopy and 35 samples that were missed by LED-FM microscopy. Importantly, there were 31 samples that were missed by both ZN and LED-FM microscopy but were positive by BioFM-Filter microscopy. However, there were 13 and 2 samples that were missed by BioFM-Filter microscopy but were ZN smear-positive and LED-FM positive, respectively. At Site 2, a similar trend was observed in the performance of BioFM-Filter microscopy (S3 Table).

#### Performance of ‘TB Concentration & Transport’ and ‘TB DNA Extraction’ kits

**Fig 8:**
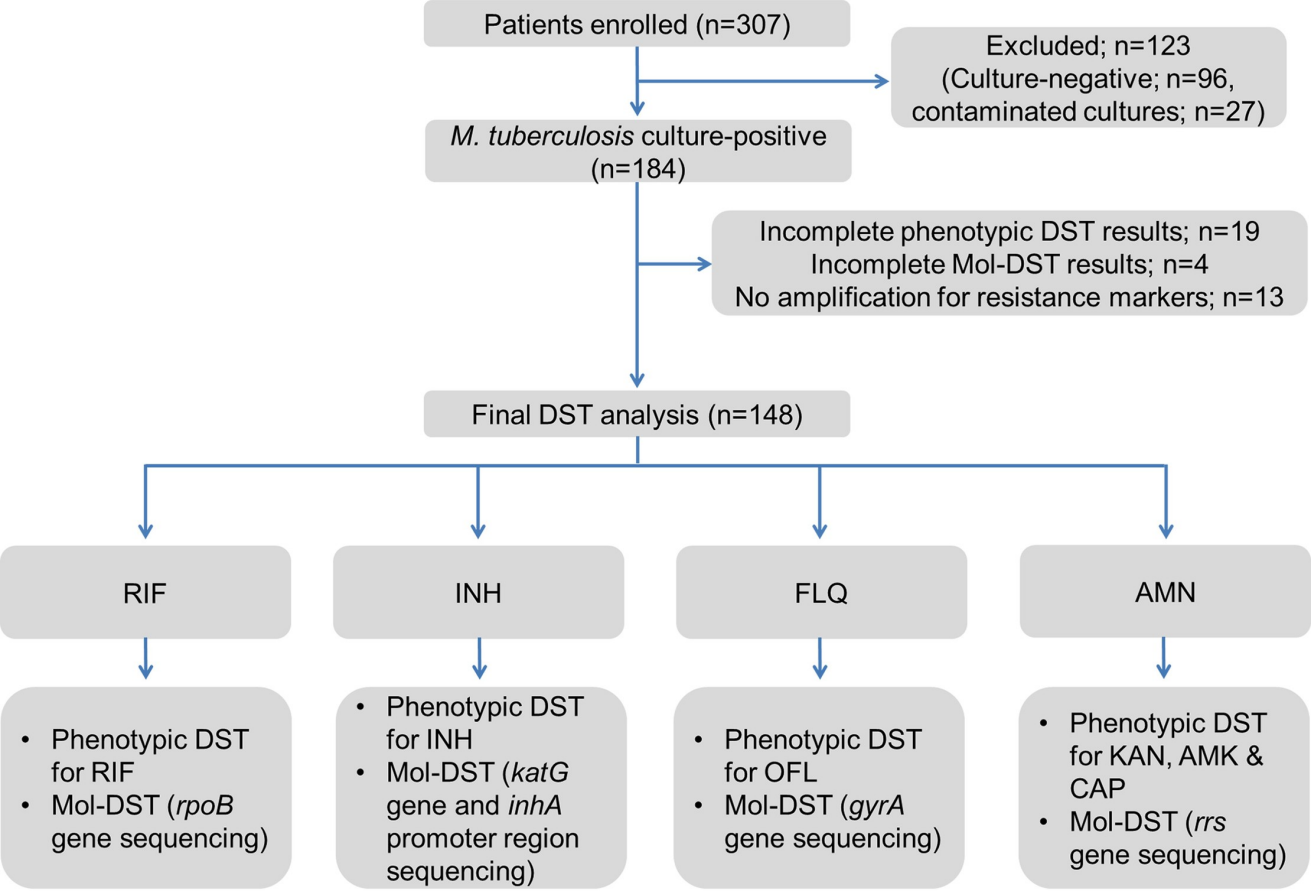
Participant enrolment and testing in ‘TB concentration & transport’ kit and ‘TB DNA extraction’ kit evaluation study.

A total of 307 sputum samples were analyzed in this study, out of which 159 samples were excluded (Figs 3C and 8). The performance of Mol-DST was compared to that of phenotypic DST in 148 culture-positive samples. Notably, 32 of these 148 culture-positive samples were smear–negative and drug resistance profiles were generated from these samples using Mol-DST assay. Amongst these 148 samples, 81 were scored as sensitive and 45 as resistant by both RIF phenotypic DST and Mol-DST (Table 2). For INH, 74 were sensitive and 52 were resistant by both phenotypic DST and Mol-DST. In FLQ-DST analysis, 102 were sensitive and 29 were resistant by both assays and for AMN, 129 were sensitive and 6 were resistant by both phenotypic DST and Mol-DST (Table 2). Discrepant samples in each category are summarized in Table 2. In summary, the overall sensitivity of Mol-DST for detecting drug resistance ranged between 75% to 90% and the specificity ranged between 88 to 93%. Taking together these observations, a concordance of ~89 to 92.4% (κ value; 0.8–0.9) was noted between Mol-DST and phenotypic DST for all the drugs (Table 2).

**Table 2:** Performance of Mol-DST vs. phenotypic DST^#^.

Drug^a^	Mol-DST + Phenotypic DST*				Sensitivity^b^		Specificity^b^		Concordance (κ coefficient)
	**R+R**	**R+S**	**S+R**	**S+S**	**No./Total**	**% (95% CI)**	**No./Total**	**% (95% CI)**	
**RIF**	45	11	5	81	45/50	90.0 (78–96)	81/92	88.0 (79–94)	88.7 (0.8)
**INH**	52	6	10	74	52/62	83.9 (72–92)	74/80	92.5 (84–97)	88.7 (0.8)
**FLQ**	29	10	6	102	29/35	82.9 (66–93)	102/112	91.1 (84–95)	89.1 (0.8)
**AMN**	6	9	2	129	6/8	75.0 (35–97)	129/138	93.5 (88–97)	92.4 (0.9)

^#^Samples that showed mixed infection by Mol-DST were excluded in this analysis

*R- Drug resistant, S- Drug sensitive

^a^ RIF- Rifampicin, INH- Isoniazid, FLQ- Fluoroquinolones, AMN- Aminoglycosides

^b^ Sensitivity and specificity of detecting drug resistance

#### Mutations associated with drug resistance

The Mol-DST assay provides information on various mutations that contribute to drug resistance. Thus in RIF resistant samples, the most commonly observed mutations were S531L (n = 37), followed by L533P (n = 6). In INH resistant samples, S315T in the *katG* gene was the most frequent mutation (n = 45), followed by T-8C (n = 4) and C-15T (n = 4) in the *inhA* promoter region. In FLQ resistant samples, the most common mutations observed were D94G (n = 10) followed by D94A (n = 6) in *gyrA* gene. A silent mutation S95T (a known silent polymorphism) was found in a majority of samples (n = 119/148). In AMN resistant samples, A1401G nucleotide change in *rrs* gene was observed (n = 4), as the most common mutation in this study.

An added advantage of the Mol-DST assay was the detection of 15 heteroresistant samples (mixed genotype) among samples that were classified as drug sensitive by phenotypic DST. Electropherogram analysis revealed the presence of two distinct peaks at the mutation hotspot, indicating the presence of both wild type and mutant bacteria (S1 Fig). The heteroresistant samples included 6 samples that showed a mixed genotype for RIF, 6 for INH, 1 for FLQ and 2 for AMN (S1 Fig).

## Discussion

The kits developed in this study offer the advantages of sample disinfection and minimize aerosol formation by avoiding a centrifugation step during sputum concentration. A minimum 8-log kill of *M*. *tuberculosis* was achieved in spiked sputum samples processed by the kit method, thereby minimizing bio-hazards to technicians working in basic laboratory set-ups. The level of disinfection provided by the kits was comparable to previously published data of the ‘Sample Reagent’ (SR) in the Xpert MTB/RIF assay [37], however, no direct comparison with Xpert MTB/RIF assay was performed in the present study.

An overall 7% increment in positivity over ZN microscopy and a 4% increment in positivity over LED-FM were noted by BioFM-Filter microscopy using ‘TB Detect’ kit. The factors contributing to improved positivity include (i) using a larger sputum volume (~60 μl vs. ~20 μl), (ii) technician-friendly procedure that minimizes variability, (iii) greater convenience (kit includes all processing and staining reagents, it takes less time to read each slide owing to an enhancement in smear grade status, fewer number of fields to view and a clear background), and (iv) BioFM-Filter concentrates the sputum in an ~8.5-fold smaller area (9.5 mm diameter, ~70 mm^2^) as compared to conventional microscopy on a glass slide (~2 x ~3 cm, ~600 mm^2^). These features proportionately reduce the number of fields (~200) and time duration (30 seconds to 1 min) for examining each filter, as compared to conventional ZN smear microscopy that uses a glass slide {15,000 fields at 100x [38] and requires a time duration of 3 to 5 min per ZN slide [to observe 100 fields] [38–40] and ~2 min per FM slide [to observe 40 fields] [40]}. The effective staining time is ~ 30 seconds as compared to a glass slide where the effective staining time is 15–20 min. This is attributed to the fact that the glass slide uses diffusion principle for all steps, which is quite slow whereas the filter device allows rapid movement of staining reagents leading to efficient reactivity of staining molecules with the target bacteria. Moreover, the volume of reagents used for the process is reduced by an order of magnitude (only 80 to 250 μl) over conventional slide microscopy method, which makes the kit handy, cost-effective and rapid. Furthermore, the ‘TB Detect’ kit provides TB diagnosis for a batch of 10 samples in 1 hour. However, there were some samples that were missed by BioFM-Filter microscopy but were ZN smear-positive and/or LED-FM positive. The probable reason could be the uneven distribution of mycobacteria in the sputum aliquots used for these three microscopy methods.

A greater increase in positivity (8%) of BioFM-Filter microscopy over ZN smear microscopy was observed at TB Hospital, Ambala vs. 4% increase in positivity at NITRD, suggesting that Bio-FM-Filter microscopy is well suited to the technical skills of laboratory personnel even at district hospitals and associated DMCs. The RNTCP programme of India is promoting the use of fluorescence microscopy in most of its microscopy centres having a high load of TB cases [41] and the performance of ‘TB Detect’ suggests that it has the potential to replace existing direct smear microscopy and improve case detection.

The challenges towards fulfilling the goal of providing universal DST under the ‘National Strategic Plan for Tuberculosis Elimination’ 2017–2025 [42], are formidable; they include the limited number of existing NRLs/IRLs, high infrastructural costs, paucity of bio-containment laboratory network and skilled laboratory personnel, and stringent requirement for bio-safe transport of infectious specimens to central laboratories from widespread geographical locations. The ‘TB Concentration & Transport’ kit fulfils many of the desirable features in the target product profile (TPP) for sample transport [21]. The advantages offered by this kit include: (i) requirement for only basic laboratory facilities at the site of filter preparation, (ii) easy-to-follow procedure for a laboratory technician at PHCs and associated DMCs to prepare *Trans*-Filter without the use of any equipment, and (iii) transportation of dried bio-safe concentrated sputum on *Trans*-Filter at ambient temperature. The kit is also robust and operationally friendly: (i) the generated plastic waste is disposable according to TB programme guidelines, (ii) the filters and *M*. *tuberculosis* DNA on filters are stable at extreme temperatures upto 50°C for upto 30 days and (iii) DNA is rapidly isolated from filters in 1 hr and 30 min from a batch of 10 samples. Although this kit does not address the criterion for maintaining *M*. *tuberculosis* viability as mentioned in the TPP for sample transport, it is compatible with molecular detection of *M*. *tuberculosis* and drug resistance.

The results of Mol-DST were directly obtained from sputum using *Trans*-Filter and targeted DNA sequencing in 3–5 days (excluding shipping time), and was significantly faster than phenotypic DST which takes as long as 6–8 weeks including culturing time to provide results. Importantly, DNA isolated from *Trans*-Filter has the potential to integrate with LPA and other NAATs, and thereby expanding the scope of DST to samples transported from the nation-wide network of DMCs (>14000) to regional NRLs (n = 6), IRLs (n = 31) and other central and DST laboratories (n = 37). We believe the use of *Trans*-Filter technology will help to close the existing gaps in DST testing described above. Most importantly it is patient friendly, and will obviate the requirement for TB patients to visit a DST facility [43] and thereby favourably impact the initiation of anti-tubercular treatment in patients at remote geographical locations.

The diagnostic accuracy of Mol-DST assay was comparable to direct DNA sequencing from sputum [44–46] and the assay described recently by Xie et al. [47] (S4 Table). However, the diagnostic accuracy of Mol-DST was slightly lower in comparison to Xpert [48] and LPA [49, 50] (S4 Table). Even so, we believe that the ‘TB Concentration & Transport’ kit is a unique technology that combines ambient temperature transport of inactivated sputum with molecular detection of drug resistance.

A few discordant results were observed between phenotypic DST and Mol-DST tests. The discordant ‘phenotypic R’/ ‘Mol-DST Wt’ results ranged between ~1.4% to ~6.8% for AMN, RIF, FLQ and INH. One possible explanation for this discordance could be that we have not assessed the occurrence of less frequently encountered mutations, such as mutations located outside the RRDR region for RIF resistance [22], in *inhA*, *ahpC*, *mabA*, *ndh* (for INH resistance) [24], *gyrB* (for FLQ resistance) [51] and *eis*, *tlyA*, *gidB* (for AMN resistance) genes that have contributed to drug resistance in other studies [26]. Another category of discordant results was ‘phenotypic S’/ ‘Mol-DST Mut’ samples that ranged between ~4.1% to ~7.4% for INH, AMN, FLQ and RIF. One possible reason for this discordance is the use of drug concentrations in phenotypic DST that do not detect low level phenotypic resistance which is detected by Mol-DST. For example, in RIF discrepant samples, 3 samples had a L533P mutation which confers low level resistance [52, 53]. The discordance between phenotypic and genotypic DST results observed in our study as well as by others [47, 54, 55] suggests a need to redefine the drug concentrations to be tested in phenotypic DST [56–58]. The second possible reason for discordance is the occurrence of heteroresistance or mixed infection. The ability of Mol-DST to detect heteroresistance or mixed infection highlights the advantage of this method over phenotypic methods and has clinical relevance for prescribing appropriate therapy [59, 60]. Another advantage of Mol-DST assay is the detection of natural polymorphisms in genes that determine drug resistance. In our study, the S95T mutation was found in 119 of 148 samples, which has been reported earlier as a natural polymorphism [25, 61]. It is also noteworthy that we could obtain drug resistance data from 32 smear-negative culture-positive samples in this study. These findings indicate that *Trans*-Filter Mol-DST can be applied to samples that are negative by smear microscopy. This study opens up opportunities for evaluation of the kits at multi-centric sites and under programme conditions but it has some limitations. The stability of DNA on *Trans*-Filter during prolonged exposure to high temperatures was adequately confirmed under laboratory conditions; however, there is a requirement for further validation of the transport filter in field settings.

## Conclusions

The implementation of ‘near-patient’ technologies described in this study for improved smear microscopy and rapid molecular detection of TB and associated drug resistance have the potential to bridge the gap of ‘missing millions’ and improve TB control in the community. ‘TB Detect’ and ‘TB Concentration & Transport’ kits are competitively priced at <USD 1.4–1.5 per sample as compared to the cost of direct smear microscopy (USD 0.83) [62], or the available sputum transportation kits (such as PrimeStore Molecular Transport Medium [Longhorn Vaccines and Diagnostics LLC, US, ~USD 12/sample, https://www.lhnvd.com/product-page/primestore-mtm] and OMNIgene SPUTUM [DNA Genotek, Canada, USD 1.15/ml of sputum]) [21], and commercial DNA extraction kits (USD 3 to 4 per sample). We believe that this innovative equipment-free bio-safe technology successfully addresses several of the unmet challenges faced while diagnosing TB and drug resistance in high burden resource-limited countries, including India. Finally, the development and evaluation of the kits have paved the way for large-scale field studies of ‘TB Detect’, ‘TB Concentration & Transport’ and ‘TB DNA Extraction’ kits to assess their utility and impact in high TB burden settings.

## Overall work distribution

The academic institutions (AIIMS and THSTI) and industry partner (Advanced Microdevices Pvt Ltd ‘mdi’ Ambala, India) were involved in the development of solutions (‘Dissolving solution’ and ‘Sterilizing solution’ and others) and ‘Filtration device’. ‘mdi’ assembled all the components into kits which were provided to NITRD Hospital, TB Hospital Ambala and THSTI for testing; which involved the assessment of smear microscopy by the ‘TB Detect’ kit and sputum transport by the ‘TB Concentration & Transport’ kit and subsequent use of extracted DNA using the ‘TB DNA Extraction’ kit for Molecular Drug susceptibility Testing (Mol-DST). AIIMS and THSTI co-ordinated the study with mdi. All the authors are joint inventors in an Indian Provisional Patent application named ‘Apparatus and method for processing a sample for rapid diagnosis of tuberculosis and safe transport of bacteria’ (Patent application number- 201811042155).

## Supporting information

S1 Appendix Patient consent form from hospital sites (NITRD and TB hospital, Ambala). (PDF)

S2 Appendix STARD checklist. (PDF)

S3 Appendix Standardization experiments. (PDF)

S1 Fig Representative electropherogram for hetero-resistant samples. (**A**) In *rpoB* gene (at codon 531) wild type (TCG) and mutant (TTG), (**B**) In *inhA* promoter region (at upstream nucleotide 24) wild-type (G) and mutant (C), (**C)** In *gyrA* gene at (codon 94), wild type (GAC) mutant (GGC), (**D)** In *rrs* gene (at nucleotide 1401) wild-type (A) and mutant (G), mixed infection.
(PDF)

S1 Table Clinical characteristics of enrolled participants in the present study. (DOCX)

S2 Table Evaluation of sputum disinfection by ‘TB Detect’ and ‘TB concentration & transport’ kits. (DOCX)

S3 Table Positivity of BioFM-Filter, ZN and LED-FM microscopy. (DOCX)

S4 Table Comparison of the diagnostic accuracy of Mol-DST and other tests. (DOCX)

## Abbreviations

AMK: Amikacin
AMN: Aminoglycosides
BioFM-Filter: Bio-safe fluorescent microscopy filter
CAP: Capreomycin
DMCs: Designated Microscopy Centres
FLQ: Fluoroquinolones
INH: Isoniazid
IRLs: Intermediate Reference Laboratories
KAN: Kanamycin
LED-FM: Light-emitting diode fluorescence microscopy
LEV: Levofloxacin
LOD: Limit of detection
LPA: Line Probe Assay
MDR-TB: Multi drug resistant tuberculosis
MGIT: Mycobacteria Growth Indicator Tube
Mol-DST: Molecular-drug susceptibility testing
MOX: Moxifloxacin
Mut: Mutant
M. tuberculosis: Mycobacterium tuberculosis
NALC: N-Acetyl-L-Cysteine
NRLs: National Reference Laboratories
OFL: Ofloxacin
PCR: Polymerase Chain Reaction
PHCs: Primary Healthcare Centres
Phenotypic R: Phenotypic-resistant
Phenotypic S: Phenotypic-sensitive
PBS: Phosphate buffer saline
POC: Point-of-care
QRDR: Quinolone Resistance Determining Region
RIF: Rifampicin
RNTCP: Revised National TB Control Program
RRDR: Rifampicin Resistance Determining Region
STARD: Standards for Reporting of Diagnostic Accuracy
*Trans*-Filter: Transport filter
TB: Tuberculosis
WHO: World Health Organization
Wt: Wild-type
XDR-TB: Extensively drug-resistant tuberculosis
ZN: Ziehl Neelsen
