# Supplementary material for: Development and evaluation of novel bio-safe filter paper-based kits for sputum microscopy and transport to directly detect Mycobacterium tuberculosis and associated drug resistance
Source: PLoS One. 2019 Aug 13;14(8):e0220967. doi: 10.1371/journal.pone.0220967 (PMC6692035; doi:10.1371/journal.pone.0220967)
Supplement: S1 Appendix — (PDF) [file pone.0220967.s001.pdf]

S1 Appendix: Patient Consent Form

NITRD, New Delhi

Title: “Novel Sample Processing for the Simple and Rapid Diagnosis of TB, MDR-TB and XDR-TB”.

I ____ have been explained, in my own language, about the said research work **“Novel Sample Processing for the Simple and Rapid Diagnosis of TB, MDR-TB and XDR-TB”** at the National Institute of Tuberculosis and other Respiratory Diseases (NITRD), New Delhi and I gave full consent for my/my ward’s participation in this project. I also consent for collecting sputum samples and allowing procedures involved in this research. The data collection from this study shall be kept confidential. I am well informed about the details of routine investigations and will get the result of the same. The examining doctor and his/her team, will be allowed to through my/my ward’s medical and research records related to this study. The data from this study may be published in Journals or Books. However, my/my ward’s identity will not be disclosed until required by law. The study results will be informed to me as well as the treating physician for further management and patients care.

It has been further informed to me that my/my ward’s participation in this study shall be based on my willful consent and I can withdraw my/my ward’s participation at any point of time during the study for which I shall not be liable for any penalty or loss of those benefits which I deserve. I have also been informed that the investigator (doctor) can withdraw my participation from the study at any time.

All my queries have been answered to my satisfaction. I have thoroughly gone through the information written above. I have decided to participate in his study at own will. After signing on this form I shall receive a copy of the same.

Name of the Patient ________________________________

Signature of the Patient/ Parent ______________________________

Name of the witness ________________________________

Signature of the witness ______________________________

In case of any queries or emergency contact:

Room No. 113,

National Institute of Tuberculosis & Respiratory Diseases

Sri Aurobindo Marg, New Delhi – 110030

Phone: 26517829, 26517830 Ext. 113

Patient Consent Form: TB Hospital, Ambala

Title: "Novel Sample Processing for the Simple and Rapid Diagnosis of TB, MDR-TB and XDR-TB".

I ____ have been explained, in my own language, about the said research work **"Novel Sample Processing for the Simple and Rapid Diagnosis of TB, MDR-TB and XDR-TB"** at the TB Hospital, Ambala and I gave full consent for my/my ward's participation in this project. I also consent for collecting sputum samples and allowing procedures involved in this research. The data collection from this study shall be kept confidential. I am well informed about the details of routine investigations and will get the result of the same. The examining doctor and his/her team, will be allowed to through my/my ward's medical and research records related to this study. The data from this study may be published in Journals or Books. However, my/my ward's identity will not be disclosed until required by law. The study results will be informed to me as well as the treating physician for further management and patients care.

It has been further informed to me that my/my ward's participation in this study shall be based on my willful consent and I can withdraw my/my ward's participation at any point of time during the study for which I shall not be liable for any penalty or loss of those benefits which I deserve. I have also been informed that the investigator (doctor) can withdraw my participation from the study at any time.

All my queries have been answered to my satisfaction. I have thoroughly gone through the information written above. I have decided to participate in his study at own will. After signing on this form I shall receive a copy of the same.

Name of the Patient ______________________________

Signature of the Patient/ Parent ___________________________

Name of the witness ______________________________

Signature of the witness ___________________________

In case of any queries or emergency contact:

Dr. Hitesh Verma

TB Hospital Ambala

Contact no. 9467003812
