# Supplementary material for: Development and evaluation of novel bio-safe filter paper-based kits for sputum microscopy and transport to directly detect Mycobacterium tuberculosis and associated drug resistance
Source: PLoS One. 2019 Aug 13;14(8):e0220967. doi: 10.1371/journal.pone.0220967 (PMC6692035; doi:10.1371/journal.pone.0220967)
Supplement: S2 Appendix — (PDF) [file pone.0220967.s002.pdf]

S2 Appendix. STARD checklist.

Section & Topic	No	Item	Reported on page #
**TITLE OR ABSTRACT**	**1**	Identification as a study of diagnostic accuracy using at least one measure of accuracy (such as sensitivity, specificity, predictive values, or AUC)	Page # 1and 3 The title and abstract identifies the manuscript as a study of diagnostic accuracy.
**ABSTRACT**	**2**	Structured summary of study design, methods, results, and conclusions (for specific guidance, see STARD for Abstracts)	Page # 3 A structured abstract including Background, Methods and Findings, and Conclusions is included.
**INTRODUCTION**	**3**	Scientific and clinical background, including the intended use and clinical role of the index test	Page # 4 to 5 The Introduction focuses on the manuscript in a wider context. It includes a brief review of the key references and the need for the development of the 3 kits namely 'TB Detect', 'TB Concentration & Transport' and 'TB DNA Extraction' kits as described. It explains the relevant collaborations undertaken to implement and undertake the study.
	**4**	Study objectives and hypotheses	Page # 5 to 6 Towards aligning with the TB Elimination goals in high burden settings in a cost-effective manner, 3 kits were developed, namely 'TB Detect', 'TB Concentration & Transport' and 'TB DNA Extraction' kits. The

			manuscript involves the development and evaluation of these novel equipment-free, self-contained kits for TB diagnosis and associated drug resistance.
**METHODS**			
Study design	**5**	Whether data collection was planned before the index test and reference standard were performed (prospective study) or after (retrospective study)	Page # 6<br>Data collection was planned before the index test and reference standard were performed. The study was a prospective study.
Participants	**6**	Eligibility criteria	Page #8, 10 to 11<br>Patients belonging to either the Presumptive TB group (for evaluation of the ‘TB Detect’ kit) or Presumptive MDR-TB/XDR-TB group (for the evaluation of the ‘TB Concentration & Transport’ and ‘TB DNA Extraction’ kits) were included in the study.
	**7**	On what basis potentially eligible participants were identified<br>(such as symptoms, results from previous tests, inclusion in registry)	Page #8, 10 to 11 and S1 Table<br>Patients belonging to either the Presumptive TB group (for evaluation of the ‘TB Detect’ kit) or Presumptive MDR-TB/XDR-TB group (for the evaluation of the ‘TB Concentration & Transport’ and ‘TB DNA Extraction’ kits) were included in the study. Symptoms are described in S1 table.
	**8**	Where and when potentially eligible participants were identified (setting, location and dates)	Page #6, 10 to 11<br>All samples were collected between August 2016 to August 2017 after obtaining ethical clearance from the Institutional Ethics Committees of National

			Institute of Tuberculosis and Respiratory Diseases (NITRD, EC/LRS/2013/2543), TB Hospital, Ambala (BIRAC/2015/9), All India Institute of Medical Sciences (IEC/NP-135/2013) and Translational Health Science and technology Institute [THS 1.8.1 (14)]. We obtained written informed consent from participants in accordance with ethical guidelines from participating institutions (S1 Appendix).
	**9**	Whether participants formed a consecutive, random or convenience series	Page #8, 10 to 11 Patients belonging to either the Presumptive TB group (for evaluation of the 'TB Detect' kit) or Presumptive MDR-TB/XDR-TB group (for the evaluation of the 'TB Concentration & Transport' and 'TB DNA Extraction' kits) were included consecutively in the study.
Test methods	**10a**	Index test, in sufficient detail to allow replication	Since our study involves development and evaluation of kits, we have given details of the kit(s) protocol in page number 11 and 13 which can be sufficiently replicated while using the kit manual/protocol.
	**10 b**	Reference standard, in sufficient detail to allow replication	Page #12 Culture was used as a reference standard wherever applicable, which is the conventional gold standard for TB diagnosis. Details explained in referenced

			page numbers.
	**11**	Rationale for choosing the reference standard (if alternatives exist)	NA
	**12a**	Definition of and rationale for test positivity cut-offs or result categories of the index test, distinguishing pre-specified from exploratory	Page #11 to 13<br>The TB Detect' kit was assessed by its endpoint i.e. visualising TB bacteria under the microscope. The 'TB Concentration & Transport' and 'TB DNA Extraction' kits were assessed by the ability to recover DNA and perform subsequent Molecular-DST from the transported filter.
	**12 b**	Definition of and rationale for test positivity cut-offs or result categories of the reference standard, distinguishing pre-specified from exploratory	Page #11 to 13<br><br>MGIT Culture was performed by standard NALC-NaOH method. The results were confirmed by using ZN smear and SD BIOLINE TB Ag MPT64 Rapid test (Standard Diagnostics).
	**13a**	Whether clinical information and reference standard results were available to the performers/readers of the index test	Page #10 to 13<br>The clinical information and reference standard results were not available to the performers/readers of the index test as the study was carried out in a double-blind manner.
	**13 b**	Whether clinical information and index test results were available<br>to the assessors of the reference standard	Page #10 to 13<br>The clinical information and index test results were not available<br>to the assessors of the reference standard as the study was carried out in a double-blind manner.
Analysis	**14**	Methods for estimating or comparing measures of diagnostic accuracy	Page #13<br>Included in statistical analysis.
	**15**	How indeterminate index test or reference	Page # 16 to 17, Fig 8

		standard results were handled	Samples with indeterminate results were excluded from the study.
	**16**	How missing data on the index test and reference standard were handled	Page # 23 to 24, Fig 8 Samples with indeterminate/missing results were excluded from the study.
	**17**	Any analyses of variability in diagnostic accuracy, distinguishing pre-specified from exploratory	Page #13 to 14 included in statistical analysis
	**18**	Intended sample size and how it was determined	Page #8, 10 to 11, Fig 4 Fig 5 and S1 Table Included in 'Selection of subjects' section.
**RESULTS**			
Participants	**19**	Flow of participants, using a diagram	Page #8, 10 and 11, Fig 4 and Fig 5
	**20**	Baseline demographic and clinical characteristics of participants	Page # 21 to 23 and S2 Table
	**21a**	Distribution of severity of disease in those with the target condition	NA
	**21 b**	Distribution of alternative diagnoses in those without the target condition	NA
	**22**	Time interval and any clinical interventions between index test and reference standard	NA
Test results	**23**	Cross tabulation of the index test results (or their distribution) by the results of the reference standard	Page # 16 to 18 (Table 1 and Table 2)
	**24**	Estimates of diagnostic accuracy and their precision (such as 95% confidence intervals)	Page # 16 to 18 (Table 1 and Table 2)
	**25**	Any adverse events from performing the index test or the reference standard	NA
**DISCUSSION**			
	**26**	Study limitations, including sources of potential bias, statistical uncertainty, and generalisability	Page # 23 and 24 Included in discussion section
	**27**	Implications for practice, including the intended use and clinical role of the index test	Page # 19 to 22 and 24<br><br>The 'near-patient' technologies described in the manuscript provide improved smear microscopy and

			transportation solutions for the rapid detection of TB and associated drug resistance.<br>Other details are included in discussion section.
**OTHER INFORMATION**			
	**28**	Registration number and name of registry	NA
	**29**	Where the full study protocol can be accessed	NA
	**30**	Sources of funding and other support; role of funders	Uploaded on journal site
