# Supplementary material for: Development and evaluation of novel bio-safe filter paper-based kits for sputum microscopy and transport to directly detect Mycobacterium tuberculosis and associated drug resistance
Source: PLoS One. 2019 Aug 13;14(8):e0220967. doi: 10.1371/journal.pone.0220967 (PMC6692035; doi:10.1371/journal.pone.0220967)
Supplement: S3 Appendix — (PDF) [file pone.0220967.s003.pdf]

S3 Appendix

Towards developing a robust sputum liquefaction protocol, various versions of the 'Dissolving solution' (S1) were tested with a variety of sputum samples and assessed for its liquefaction capacity and to assess BioFM-Filter's filterability, and compatibility with in-situ fluorescence. The details of various versions (Ver) are summarized in Table A1.

Table A1. Experiments performed to optimize and validate Dissolving solution and BioFM-Filter.

**Dissolving solution (S1) version (Ver)**	**No. of sputum samples tested**	**Types of sputum (quality)**	**Protocol for optimization**	**Results**	**Comments based on stability of 'Dissolving solution' and *in-situ* fluorescence staining**	**Conclusion**
Ver 1.0	n=118	1.Thick<br>2.Purulent (Yellowish mucus)<br>3.Saliva	Dissolving solution and sputum in ratio of 1:2	BioFM-Filter smear showed a lower positivity of 6.8% as compared to LED-FM smear (28%). The filterability of the liquefied sputum was not 100%.	Solution stable in terms of phase separation. As compared to LED-FM, the bacteria were faintly visible after in-situ staining with increased background.	The solution was not as effective in terms of fluorescence microscopy and sputum liquefaction.
Ver 2.0	n=50	1.Thick<br>2.Purulent (Yellowish mucus)<br>3.Saliva	Dissolving solution and sputum in ratio of 1:2	BioFM-Filter smear showed a positivity of 46% as compared to LED-FM smear (54%). The filterability of the liquefied sputum was not 100%.	Solution stable in terms of phase separation. As compared to LED-FM, the bacterial visibility after in-situ staining was improved (appeared as	Improved visibility in BioFM-Filter smear microscopy and so does sputum liquefaction. But, it still needed further improvement.

					bright rod shaped) with decreased background.	
Ver 3.0	n=155	1.Thick 2.Purulent (Yellowish mucus) 3.Saliva	Dissolving solution and sputum in ratio of 1:4	BioFM-Filter smear showed an improved positivity of 26.4% as compared to LED-FM smear (25.8%). The filterability of the liquefied sputum was 100%.	Solution stable in terms of phase separation and also for 6 months at room temperature conditions. The bacterial visibility after in-situ staining was comparable to LED-FM (as bright rod shaped) with no background.	BioFM-Filter smear microscopy using this solution was better than LED-FM microscopy. This was thus finalized as the final Dissolving solution.

Details of studies.

Study 1.

Dissolving Solution ver 1.0 (S1 ver 1.0). S1 ver 1.0 was tested for LED-FM using 118 sputum specimens. S1 ver 1.0 was a formulation of Guanidinium Hydrochloride, Tris buffer, Triton X-100 and reducing agent of functionality similar to DTT. The study protocol is shown in **Fig A1**. A direct smear was prepared before sputum processing. Then, S1 ver 1.0 was added to 150 µl of sputum in a ratio of 1:2 (v/v) and incubated for 30 minutes. The liquefied sputum was added to the device and filtered. The quality of sputum and filtration characteristics of diluted sputum are shown in **Table A2.** Filtration smear microscopy showed a lower positivity of 6.8% as compared to direct smear (28%, **Table A3**) and the filterability of the liquefied sputum was also not 100% (**Table A3)**. Eight samples out of 118 samples were partially filtered (minimum 300 µl was filtered). All eight

samples that were partially filtered had a very thick sputum quality. Also, out of these eight samples, one sample had a positive direct smear status.

Conclusion. Dissolving Solution ver 1.0 was stable in terms of phase separation, however it was not very effective in terms of sputum liquefaction and BioFM-Filter based fluorescence microscopy; bacteria were faintly visible after in-situ staining with high background when compared to direct LED-FM smear microscopy.

Study 2.

Dissolving Solution ver 2.0 (S1 ver 2.0). Dissolving solution S1 ver 1.0 was modified to S1 ver 2.0 by changing the concentrations of components and addition of N-Acetyl L-Cysteine. The study design and methodology was same as that for study 1 **(Fig A1)**. A marked improvement in sputum liquefaction and visibility in smear was noted, however the BioFM-Filter smear using S1 ver 2.0 showed a positivity of 46% as compared to direct smear which yielded 54% positivity (**Table A3**). **Table A2** shows the quality of sputum and filtration characteristics of sputum. Six samples were partially filtered (thick sputum). Out of these six samples, three samples had a positive direct smear status. Partial filtration of these three samples can also be one of the reasons for the low positivity of BioFM-Filter smear as compared to direct smear.

Conclusion. Dissolving Solution ver 2.0 was stable in terms of phase separation. As compared to LED-FM, the bacterial visibility after in-situ staining was improved (bacteria appeared as bright rod-shaped structures) with decreased background. BioFM-Filter smear microscopy in terms of visibility with low background and sputum liquefaction were improved. However, it still needed further improvement.

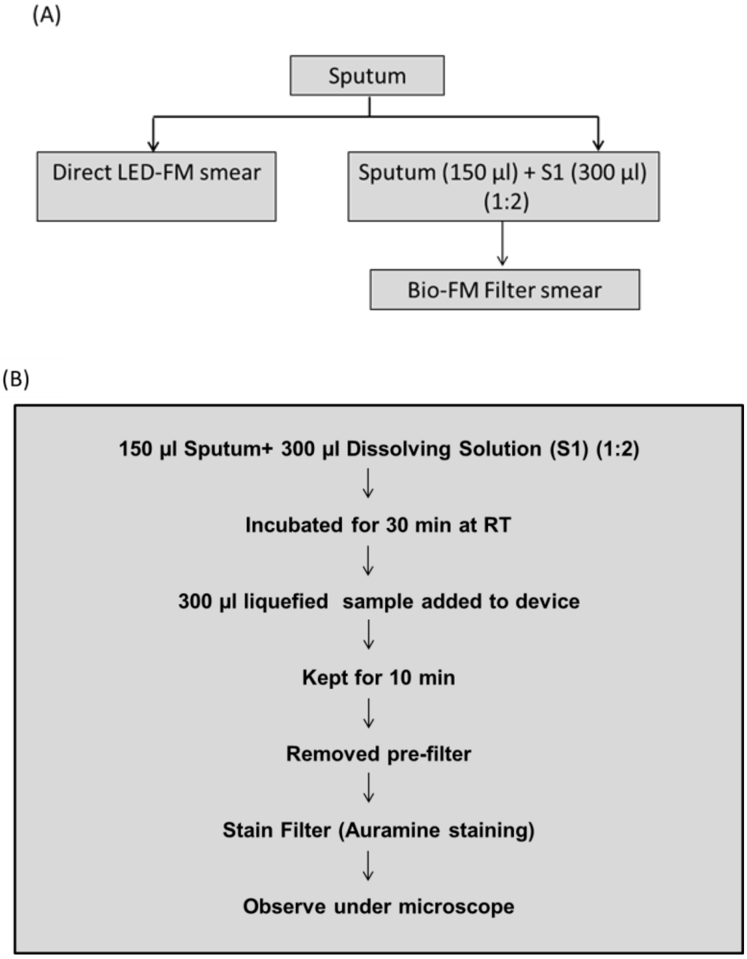

Fig. A1. (A). Design of study 1 and study 2; **(B).** Processing protocol for BioFM-Filter smear microscopy.

Table A2. Quality and filtration characteristics of liquefied sputum using S1 ver 1.0 (n=118) and S1 ver 2.0 (n=50).

**Filtration of liquefied sputum**	**S1 ver 1.0 No. of samples**	**S1 ver 2.0 No. of samples**
Complete	110	46
Partial	8	6
Total	118	50
**Sputum quality**		
Thick	26	21
Purulent (Yellowish mucus)	57	25
Saliva	35	4
**Total**	118	50

Table A3. Performance of BioFM-Filter smear using S1 ver 1.0 and S1 ver 2.0 compared to direct LED-FM smear.

**Microscopy**	**S1 ver 1.0 (n=118)**		**S1 ver 2.0 (n=50)**	
Smear Microscopy	Positive	Negative	Positive	Negative
Direct LED-FM smear	33	85	27	23
BioFM-Filter smear	8	110	23	27

<u>**Study 3.**</u>

Dissolving Solution ver 3.0 (S1 ver 3.0). S1 ver 2.0 was further modified to S1 ver 3.0 (by changing concentrations of various components) based on the observations made in study 1 and 2. S1 ver 3.0 was tested in a study of 155 sputum specimens. The study design was the same as followed for study 1 and study 2 except that 4 volumes of S1 ver 3.0 was used per volume of sputum (1:4) for liquefaction (**Fig A2**). The quality of sputum and filtration characteristics of sputum samples used with S1 ver 3.0 is shown in Table A4. The filterability of liquefied sputum is 100% with all types of sputum including thick and mucopurulent specimens **(Table A4)**. Liquefied sputum specimens were analyzed by microscopy (both direct LED-FM smear and BioFM- Filter smear). BioFM- Filter smear showed further improvement with 26.4% positivity as compared to that direct smear, which was 25.8% (**Table A5**).

Conclusion. Dissolving Solution ver 3.0 was stable in terms of phase separation for 6 months at room temperature conditions. The bacterial visibility after in-situ staining was comparable to LED-FM (as bright rod shaped) with no background. BioFM-Filter smear microscopy using this solution was better than LED-FM microscopy. Thus Dissolving Solution ver 3.0 was finalized and named as ‘Dissolving solution (S1)’.

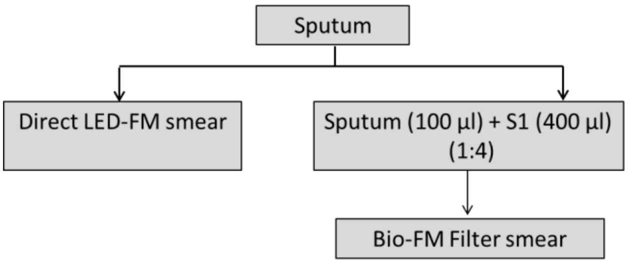

Fig. A2. Study 3 protocol.

Table A4. Quality and filtration characteristics of liquefied sputum using S1 ver 3.0 (n=155).

**Liquefied sputum filtration**	**No. of samples**
Complete	155
Partial	0
Total	155
**Sputum quality**	
Thick	75
Purulent (Yellowish mucus)	63
Saliva	17
**Total**	155

Table A5. Performance of BioFM-Filter smear using S1 ver 3.0 (n=155) compared to direct LED-FM smear.

Smear Microscopy	Positive	Negative
Direct LED-FM Smear	40	115
BioFM-Filter Smear	41	114

Development of Filtration devices (BioFM-Filter and *Trans*-Filter device).

The final Filtration devices were standardized through various versions. Several membrane materials, like polycarbonate, polytetrafloroethylene (PTFE), polyether sulphone (PES), nylon (NYL) and mixed cellulose ester (MCE) were tested for compatibility with ZN/Auramine O staining (BioFM-Filter) and bacterial retention for transport and DNA isolation (*Trans*-Filter). One of these membranes with 0.6 micron pore size was found to be most suitable for efficient sputum filtration. Filters of larger pore size (>1µ) were developed for pre-filtration to give high throughput and minimal bacterial retention.

The BioFM-Filtration device was standardized through various versions (ver 1.0 to ver 6) as shown in Fig. A3. The final locked-in device ver 6.0 was with 9 mm diameter with welded membrane and edge and slide-like base. In total 200 fields can be examined in the membrane contained in this final device at 40 × magnification.

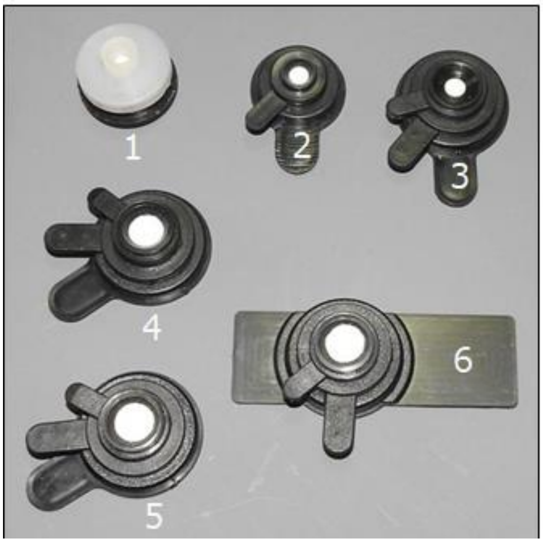

Fig. A3. Stages in the development of BioFM-Filter.

The *Trans*-Filter device was also standardized simultaneously. The Final locked-in device was made-up of various components (shown in Fig A4) with filter membrane of 12 mm diameter.

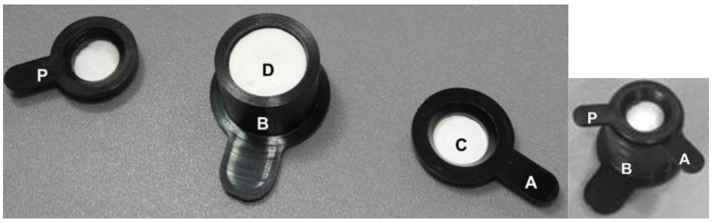

Fig. A4. Final locked-in version of *Trans*-Filter device: assembly of various components; Funnel (A), Base (B), Membrane (C), Absorbents (D) and prefilter fitted in funnel (P).

Assessment of bacterial retention on *Trans*-Filter.

To evaluate the *Trans*-Filter device for bacterial retention during transport and DNA isolation and its compatibility with PCR and sequencing, 'Dissolving solution (T1)' was used i.e. the same 'Dissolving solution (S1)' which was finalized with BioFM-Filter standardization. There was no observed difficulty in the filterability of sputum through the *Trans*-Filter device and the entire sputum volume was filtered. To assess bacterial retention on filter, the LOD of *Trans*-Filter was determined by spiking *M. tuberculosis* bacteria (H37Rv) ranging from 10,000 to 100 bacteria per ml in direct smear-negative sputum, and the spiked sputum samples were processed as described in Fig. A5. The filter obtained from the *Trans*-Filter device was utilized for DNA isolation and PCR. The bacteria were retained on *Trans*-filter upto 100 bacteria /ml sputum (Fig. 5B in main manuscript). Lower bacterial loads were not tested.

The effect of high temperature (50ºC) was assessed on bacterial retention on *Trans*-Filter device and subsequent DNA isolation and PCR. A time course study was performed with various time points (1 day, 1 week, 2 weeks, 3 weeks and 4 weeks). There was no significant decrease in the amount of DNA isolated from the *Trans*-Filter over a course of 4 weeks (Fig. 6C in the main manuscript). The same sample was assessed at all time points to minimize variability. We concluded that samples can be successfully stored and transported even at high temperatures with no adverse effect on DNA isolation and PCR.

(B)

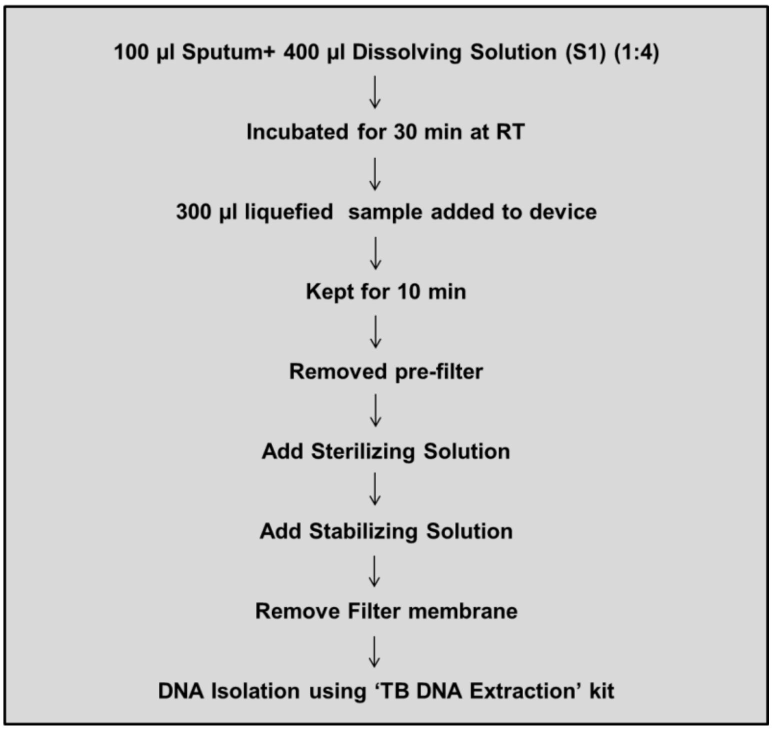

Fig A5. *Trans*-Filter study protocol.

Compatibility of *Trans*-Filter with the isolation of PCR-amplifiable DNA and sequencing from *Trans*-Filter.

The quality of DNA isolated from the *Trans*-Filter was optimal (260/280 ratio of 1.6 -1.8) and it was compatible with sequencing (Fig. A6).

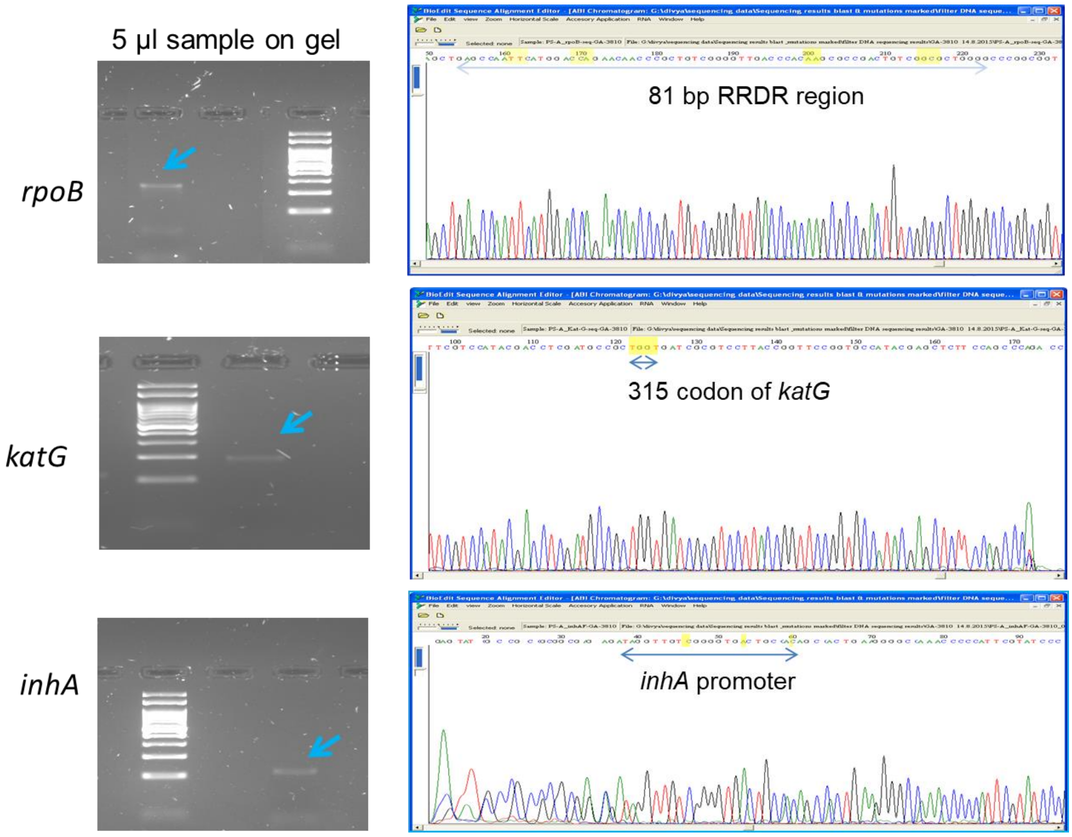

Fig A6. Sequencing results for DNA amplified from filter-extracted DNA. The isolated filter DNA was sequenced successfully by *rpoB*, *inhA* and *katG* regions for RIF and INH.

<u>Effect of 'Sterilizing Solution (T2)' on DNA isolation and PCR.</u>

DNA isolation from *Trans*-Filter device was preceded by a step of incubation with phenol based 'Sterilizing solution (T2)' to ensure that no live bacteria are present while transport of filter to the PCR laboratory. A comparison was made between DNA isolated from 'Sterilizing Solution (T2)' treated and untreated filters to ensure that the solution has no adverse effect on DNA amplification. The experiment was done in duplicate (Fig. A7). It was concluded that 'Sterilizing Solution (T2)' did not hamper the quality of DNA and subsequent amplification, indicating that it can be amalgamated successfully in all DNA isolation procedures from *Trans*-Filter device ver 2.0.

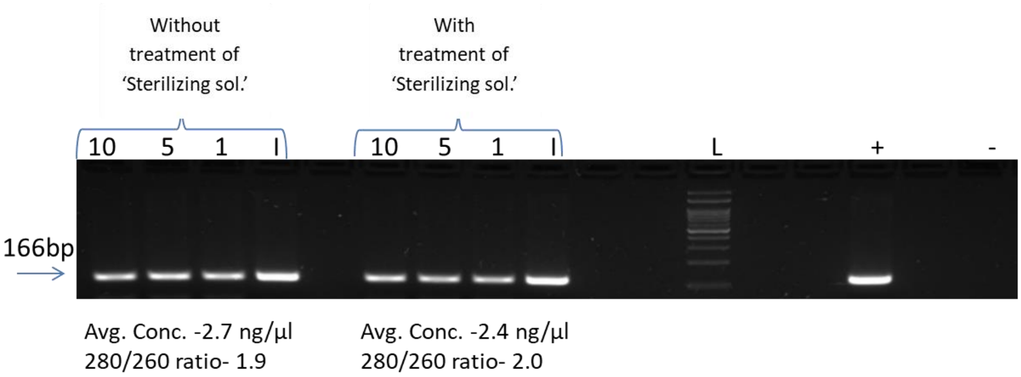

Fig. A7. Effect of sterilizing solution on DNA isolation and PCR; 10, 5, 1 denote the volume of DNA (µl) added in reaction, I is inhibitor check.

***Trans*-Filter evaluation on sputum samples.**

All sputum samples were processed as described in Fig. A5. DNA isolation and PCR was performed initially on 50 sputum samples (provided by NITRD Hospital). Representative results from 6 samples are shown in Fig. A8.

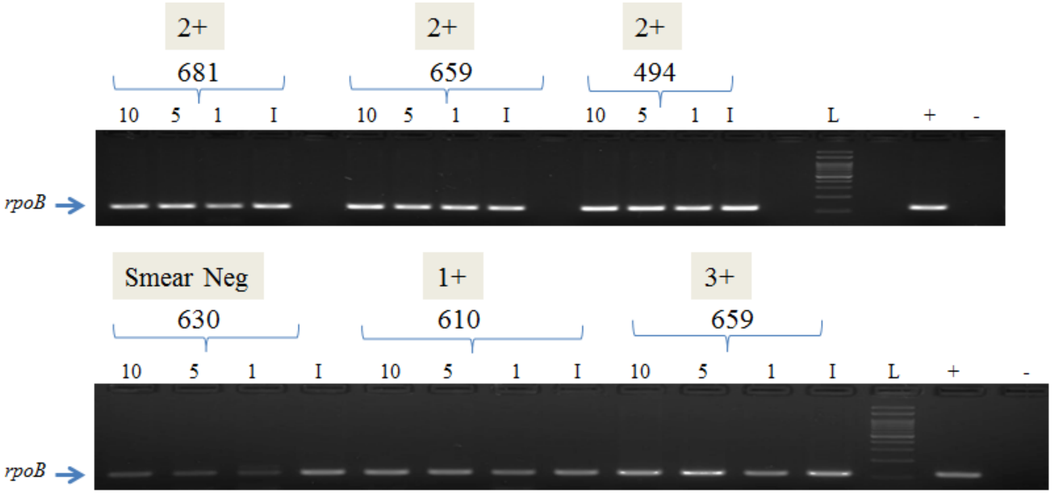

Fig. A8. Results of PCR from DNA isolated by 'TB DNA Extraction' kit sputum sample. 10, 5, 1 denote the volume of DNA (µl) added in reaction, I is inhibitor check.
