# Supplementary material for: Development and evaluation of novel bio-safe filter paper-based kits for sputum microscopy and transport to directly detect Mycobacterium tuberculosis and associated drug resistance
Source: PLoS One. 2019 Aug 13;14(8):e0220967. doi: 10.1371/journal.pone.0220967 (PMC6692035; doi:10.1371/journal.pone.0220967)
Supplement: S1 Fig — (A) In rpoB gene (at codon 531) wild type (TCG) and mutant (TTG), (B) In inhA promoter region (at upstream nucleotide 24) wild-type (G) and mutant (C), (C) In gyrA gene at (codon 94), wild type (GAC) mutant (GGC), (D) In rrs gene (at nucleotide 1401) wild-type (A) and mutant (G), mixed infection. (PDF) [file pone.0220967.s004.pdf]

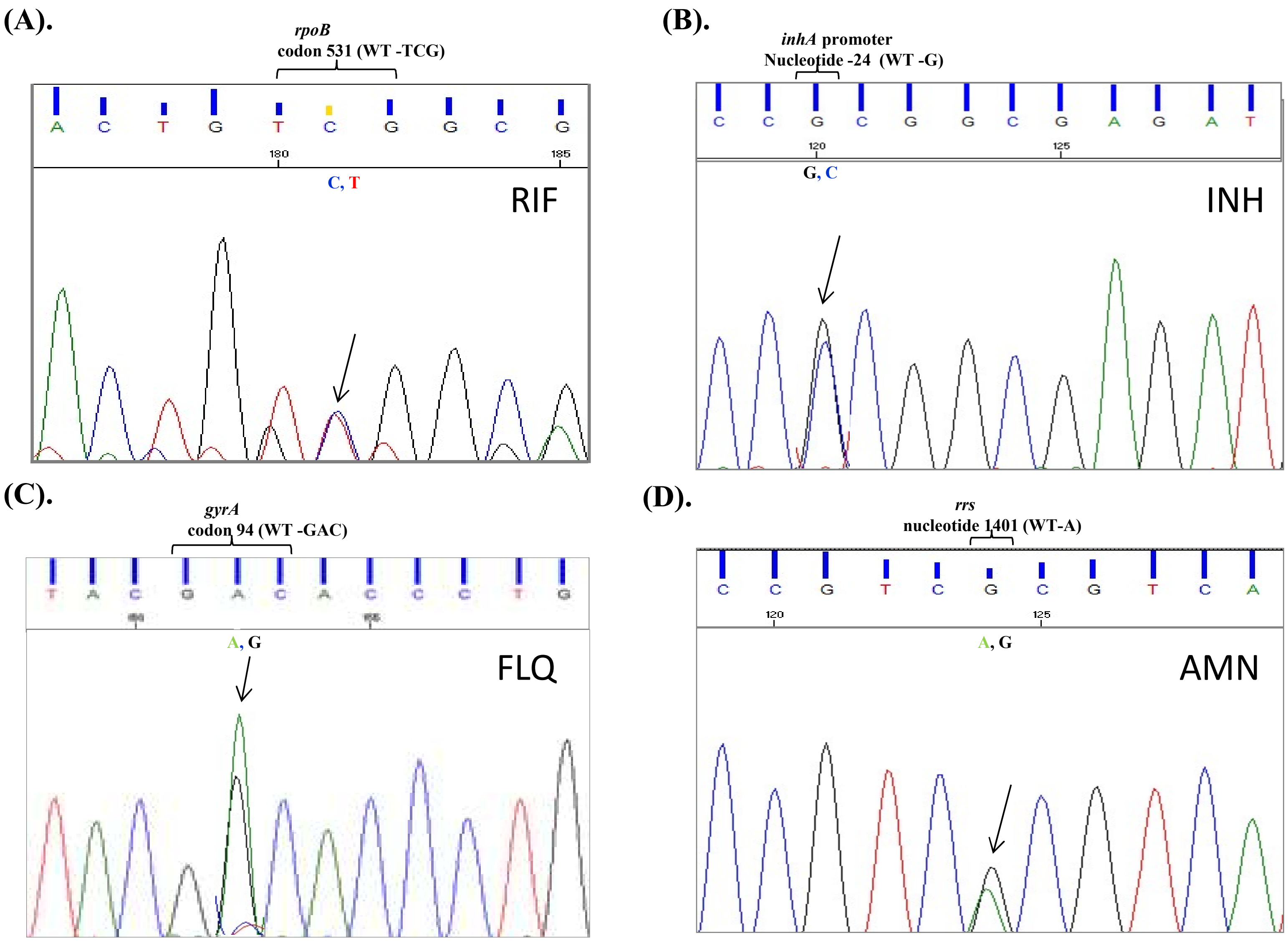

S1 Fig. Representative electropherograms for heteroresistant samples. (**A**) *rpoB* gene (codon 531) wild type (TCG) and mutant (TTG); (**B**) *inhA* promoter region (-24) wild-type (G) and mutant (C); (**C**) *gyrA* gene (codon 94), wild type (GAC) mutant (GGC), (**D**) *rrs* gene (nucleotide 1401) wild-type (A) and mutant (G).
