# Supplementary material for: Development and evaluation of novel bio-safe filter paper-based kits for sputum microscopy and transport to directly detect Mycobacterium tuberculosis and associated drug resistance
Source: PLoS One. 2019 Aug 13;14(8):e0220967. doi: 10.1371/journal.pone.0220967 (PMC6692035; doi:10.1371/journal.pone.0220967)
Supplement: S1 Table — (DOCX) [file pone.0220967.s005.docx]

**S1 Table.** Clinical characteristics of enrolled participants in the present study.

| **Symptom/ Criterion** | **Presumptive TB Patients*^$^**  **n=550 (%)** | **Presumptive**  **MDR-TB/XDR-TB Patients^#^**  **n=307 (%)** | **Total**  **n=857 (%)** |
| --- | --- | --- | --- |
| **Fever** | 528/550 (96.0) | 300/307 (97.7) | 828/857 (96.6) |
| **Cough** | 548/550 (99.6) | 280/307 (91.2) | 828/857 (96.6) |
| **Blood in sputum** | 21/550 (3.8) | 34/307 (11.0) | 55/857 (6.4) |
| **Night sweats** | 443/550 (80.5) | 280/307 (91.2) | 723/857 (84.3) |
| **Weakness** | 509/550 (92.5) | 262/307 (85.3) | 771/857 (89.9) |
| **Loss of appetite** | 451/550 (82.0) | 184/307 (59.9) | 635/857 (74.1) |
| **Weight loss** | 312/550 (56.7) | 197/307 (64.1) | 509/857 (59.4) |
| **Abdominal distension** | 263/550 (47.8) | 174/307 (56.6) | 437/857 (50.9) |
| **Diarrhoea** | 208/550 (37.8) | 127/307 (41.3) | 335/857 (39.1) |
| **Vomiting** | 109/550 (19.8) | 95/307 (30.9) | 204/857 (23.8) |
| **Lymph node enlargement** | 38/550 (6.9) | 29/307 (9.4) | 67/857 (7.8) |

*Clinical data was available only for participants enrolled at site 1 (NITRD, New Delhi)

^$^ ‘TB Detect’ kit evaluation, ^#^ ‘TB Concentration &Transport’ kit evaluation
