# Supplementary material for: Development and evaluation of novel bio-safe filter paper-based kits for sputum microscopy and transport to directly detect Mycobacterium tuberculosis and associated drug resistance
Source: PLoS One. 2019 Aug 13;14(8):e0220967. doi: 10.1371/journal.pone.0220967 (PMC6692035; doi:10.1371/journal.pone.0220967)
Supplement: S2 Table — (DOCX) [file pone.0220967.s006.docx]

**S2 Table.** Evaluation of sputum disinfection by ‘TBDetect’ and ‘TB Concentration & Transport’ kits.

| **Smear grade status of sputum** | **‘TBDetect’ kit**  **(BioFM-Filter)**  **(n= 135)** | **‘TB Concentration & Transport’ kit**  **(*Trans*-Filter)**  **(n=50)** | **Sputum culture*** | **Culture from kit filter (Turbidity)**** |
| --- | --- | --- | --- | --- |
| **3+** | 61 | 18 | Positive | No growth |
| **2+** | 29 | 13 | Positive | No growth |
| **1+** | 34 | 7 | Positive | No growth |
| **Scanty** | 11 | 12 | Positive | No growth |

*NALC-NaOH processed sputum sample, culture positivity was confirmed by positive AFB smear and positive SD BIOLINE Ag MPT64 Rapid test.

**Negative AFB smear and negative SD BIOLINE TB Ag MPT64 Rapid test confirmed sample disinfection by kit processing.
