# Supplementary material for: Development and evaluation of novel bio-safe filter paper-based kits for sputum microscopy and transport to directly detect Mycobacterium tuberculosis and associated drug resistance
Source: PLoS One. 2019 Aug 13;14(8):e0220967. doi: 10.1371/journal.pone.0220967 (PMC6692035; doi:10.1371/journal.pone.0220967)
Supplement: S3 Table — (DOCX) [file pone.0220967.s007.docx]

**S3 Table**. Positivity of BioFM-Filter, ZN and LED-FM microscopy.

|  | **B+ Z+ L+*** | **B+ Z+** | **B+ L+** | **Z+ L+** | **B+ Z-** | **B+ L-** |
| --- | --- | --- | --- | --- | --- | --- |
| **Site 1**  **(NITRD, New Delhi)**  **(n=550)** | 127 | 131 | 133 | 129 | 37 | 35 |
| **Site 2**  **(TB Hospital, Ambala)**  **(n=640)** | 79 | 83 | 106 | 79 | 56 | 28 |
| **Combined**  **(n=1190)** | 206 | 214 | 239 | 208 | 93 | 63 |

*B- BioFM-Filter microscopy; Z- ZN smear microscopy; L- LED-FM smear microscopy

‘+’ positive; ‘-’ negative
