# Supplementary material for: Development and evaluation of novel bio-safe filter paper-based kits for sputum microscopy and transport to directly detect Mycobacterium tuberculosis and associated drug resistance
Source: PLoS One. 2019 Aug 13;14(8):e0220967. doi: 10.1371/journal.pone.0220967 (PMC6692035; doi:10.1371/journal.pone.0220967)
Supplement: S4 Table — (DOCX) [file pone.0220967.s008.docx]

**S4 Table.** Comparison of diagnostic accuracy of the Mol-DST assay with other tests.

| Drug^a^ | Mol-DST from  sputum (present  study; n=148) | | Xpert MTB/RIF assay (meta-analysis;  n= 2340)  (43) | | Assay reported by Xie et al.  (n=298-303)  (42) | | Genotype MTBDR*plus* assay  (meta-analysis; n=7913)  (44) | | Genotype MTBDR*sl*  assay (n=231;  smear-positive sputum)  (45) | | DNA sequencing  from sputum  [n=41; respiratory specimens (39), n=111 (40) and  n=186; smear-positive sputum (41)] | |
| --- | --- | --- | --- | --- | --- | --- | --- | --- | --- | --- | --- | --- |
|  | Sn (%) | Sp (%) | Sn (%) | Sp (%) | Sn (%) | Sp (%) | Sn (%) | Sp (%) | Sn (%) | Sp (%) | Sn (%) | Sp (%) |
| **RIF** | 90 | 88 | 94 | 98 | - | - | 96 | 98 | - | - | 91.7 ([44](#_ENREF_44))  96.2 ([45](#_ENREF_45))  96.9 ([46](#_ENREF_46)) | 100 ([44](#_ENREF_44))  94 ([45](#_ENREF_45))  98.3 ([46](#_ENREF_46)) |
| **INH** | 84 | 92.5 | - | - | 83 | 94 | 91 | 99 | - | - | 85.7 ([44](#_ENREF_44))  63.9 ([45](#_ENREF_45))  87 ([46](#_ENREF_46)) | 93.8 ([44](#_ENREF_44))  94.6 ([45](#_ENREF_45))  99.2 ([46](#_ENREF_46)) |
| **FLQ** | 83  (OFL) | 91  (OFL) | - | - | 88 (OFL)  87.6 (MOX;  0.5µg/ml)  96.2 (MOX;  2 µg/ml) | 94 (OFL)  94.3 (MOX; 0.5µg/ml)  84 (MOX; 2µg/ml) | - | - | 93  (OFL,  LEV, MOX) | 98.3  (OFL,  LEV,  MOX) | 77.5 ([46](#_ENREF_46)) | 97.2 ([46](#_ENREF_46)) |
| **AMN** | 75 (KAN, AMK,  CAP) | 93.5  (KAN/ AMK/ CAP) | - | - | 71.4 (KAN)  70.7 (AMK) | 99.4 (KAN)  99.6 (AMK) | - | - | 89  (KAN, AMK,  CAP) | 91.7  (KAN, AMK,  CAP) | - | - |

*Sn - Sensitivity, Sp - Specificity, ‘-’ indicates the investigation is outside the scope of the test, cited studies are within brackets.

^a^ RIF- Rifampicin, INH- Isoniazid, FLQ- Fluoroquinolones, AMN- Aminoglycosides, OFL: Ofloxacin, KAN- Kanamycin, AMK- Amikacin, CAP- Capreomycin, MOX- Moxifloxacin, LEV- Levofloxacin.
